# The Anti-Breast Cancer Activity of Dihydroartemisinin-5-methylisatin Hybrids Tethered via Different Carbon Spacers

**DOI:** 10.3390/molecules27227994

**Published:** 2022-11-18

**Authors:** Yanfen Yao, Hong Wang, Jing Xu, Feng Gao, Wei Cao

**Affiliations:** 1 Shandong Provincial Third Hospital, Shandong University, Jinan 250031, China; 2 State Key Laboratory of Biobased Material and Green Papermaking (LBMP), Qilu University of Technology, Jinan 250353, China; 3 Department of Nephrology, The First Affiliated Hospital of Shandong First Medical University & Shandong Provincial Qianfoshan Hospital, Shandong Institute of Nephrology, Jinan 250014, China; 4 The First Clinical College, Shandong University of Traditional Chinese Medicine, Jinan 250355, China

**Keywords:** artemisinin, isatin, hybrid molecules, breast cancer, drug resistance

## Abstract

Sixteen dihydroartemisinin-5-methylisatin hybrids **6a–c** and **7a–m** tethered via different carbon spacers were assessed for their antiproliferative activity against MCF-7, MDA-MB-231, MCF-7/ADR and MDA-MB-231/ADR breast cancer cell lines as well as cytotoxicity towards MCF-10A cells to investigate the influence of the length of carbon spacers on the activity. The preliminary results illustrated that the length of the carbon spacer was the main parameter which affected the activity, and hybrids tethered via the two-carbon linker showed the highest activity. Amongst the synthesized hybrids, the representative hybrid **7a** (IC_50_: 15.3–20.1 µM) not only demonstrated profound activity against both drug-sensitive and drug-resistant breast cancer cell lines, but also possessed excellent safety and selectivity profile. Collectivity, hybrid **7a** was a promising candidate for the treatment of both drug-sensitive and drug-resistant breast cancers and worthy of further preclinical evaluations.

## 1. Introduction

Breast cancer remains a worldwide public health dilemma and is the most common type of fatal ailment in females around the world [1,2]. Over 2.3 million new cases and 685,000 deaths from breast cancer occurred in 2020, and it is predicted to increase to over 3 million new cases and 1 million deaths by 2040 [3,4]. Diverse therapeutic strategies, inclusive of surgery, chemotherapy, endocrine therapy, targeted therapy, and immunotherapy, have been developed for the treatment of breast cancer. However, drug resistance has become a tremendous obstacle in overcoming recurrence and metastasis and is responsible for most breast cancer fatalities [5,6]. Moreover, current available anti-breast cancer agents could lead to some adverse events such as cardiotoxicity, bronchospasm, sleep disorders, and angioedema [7,8]. Hence, there is a critical need to develop novel anti-breast cancer agents to address drug resistance and side effects. 

Dihydroartemisinin (DHA), a derivative of artemisinin (ART), demonstrated many advantages compared to ART, including high water solubility, easy absorption, wide distribution, rapid excretion and metabolism, high efficiency, and low toxicity [9,10]. DHA could exert anticancer effects through various mechanisms, such as excessive reactive oxygen species (ROS) production, proliferation inhibition, inducing apoptosis, repression of tumor metastasis and angiogenesis, promotion of immune function, guide of autophagy, and endoplasmic reticulum (ER) stress [11,12]. Accordingly, DHA exhibits profound anticancer activities on a wide range of cancer types including breast cancer both in vitro and in vivo, representing a useful template for the discovery of novel anticancer chemotherapeutics.

Isatin (Indoline-2,3-dione or indole-1*H*-2,3-dione), an endogenous substance, is characterized by a borad of biological properties such as low toxicity, mutagenicity, and genotoxicity in vivo [13,14]. Isatin is a highly “privileged motif” for the target-based design and development of anticancer agents since its derivatives are potential inhibitors of lysine-specific histone demethylase 1 (LSD1), β-carbonic anhydrase, topoisomerase II, tyrosine kinase, proteases, phosphatases, tubulin, and epidermal growth factor receptor (EGFR) [15,16]. Notably, many isatin derivatives have been proved with promising activities against both drug-sensitive and multidrug-resistant breast cancer, and several isatin-based compounds have already been approved for cancer therapy [17,18]. Hence, isatin moiety has also been considered as a useful pharmacophore against breast cancer.

Hybridization of DHA and isatin into one molecule could obtain novel anti-breast cancer candidates with broader spectrum, higher efficiency, lower toxicity, as well as multiple mechanisms of action that could overcome drug resistance [19,20,21,22]. DHA-isatin hybrids have the potential in the treatment of breast cancer. As a continuous program to seek for more effective anti-breast cancer candidates, a series of DHA-5-methylisatin hybrids tethered via different carbon spacers were designed, synthesized, and the antiproliferative activities against drug-sensitive MCF-7 and MDA-MB-231 breast cancer cell lines as well as their multidrug-resistant counterparts MCF-7/ADR and MDA-MB-231/ADR were also evaluated in this study.

## 2. Results and Discussion

**Scheme 1:**
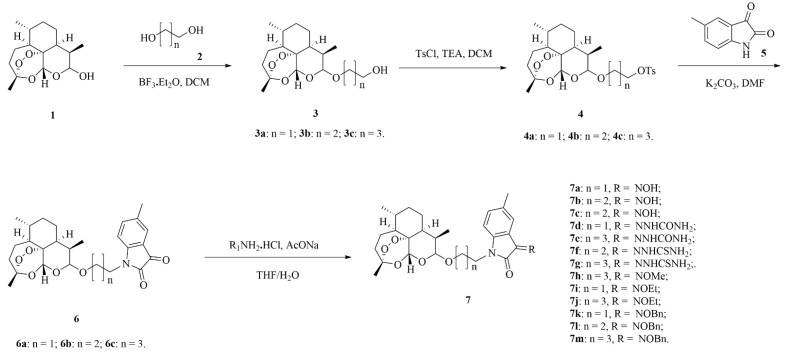
Synthetic route of DHA-5-methylisatin hybrids **6a–c** and **7a–m**.

The DHA-5-methylisatin hybrids **6a–c** and **7a–m** tethered via different carbon spacers were prepared through the synthetic route depicted in Scheme 1. 2-Hydroxyethyl/3-hydroxypropyl/4-hydroxybutyl were incorporated into C-12 position of DHA skeleton through acetalization of DHA **1** and ethylene glycol/1,3-propylene glycol/1,4-butanediol (**2**) in presence of boron trifluoride diethyl etherate (BF_3_.OEt_2_). Introduction of *p*-toluenesulfonyl (Ts) to the terminal hydroxyl group of intermediates **3** with triethylamine (TEA) as base yielded intermediates **4**, and then alkylation of 5-methylisatins with tosylates **4** provided desired DHA-5-methylisatin hybrids **6a–c**. Finally, DHA-5-methylisatin hybrids **6a–c** reacted with hydroxyamine/methoxyamine/ethoxyamine/benzyloxyamine hydrochlorides or semicarbazide/thiosemicarbazide by using sodium acetate (AcONa) as base, giving hybrids **7a–m**.

The desired DHA-5-methylisatin hybrids **6a–c** and **7a–m** tethered via different carbon spacers were characterized by high resolution mass spectrometry (HRMS), proton nuclear magnetic resonance (^1^H NMR), and carbon-13 nuclear magnetic resonance spectroscopy (^13^C NMR), and the corresponding analytical spectra were included in the Supplementary Materials. Take hybrid **6a** for an example, the characteristic signals in 1H NMR as follows: 0.75 (d, 3H), 0.97 (d, 3H), and 1.48 (s, 3H) ppm belongs to -CH_3_ at C-3, C-6, and C-9 position of DHA moiety, 4.76 (d, 1H) and 5.19 (s, 1H) belongs to C-10 and C-12 positions, respectively; 3.63–3.67 (m, 1H), 3.87–3.90 (m, 1H), 4.00–4.04 (m, 1H), and 4.14–4.18 (m, 1H) ppm belongs to the -CH_2_CH_2_- linker between DHA and 5-methylisatin; 2.36 (s, 3H) belongs to -CH_3_ at C-5 position of isatin moiety, whereas 6.86 (d, 1H), 7.38 (d, 1H) and 7.41 (s, 1H) ppm belongs to isatin moiety. For the HRMS, *m*/*z* Calcd for C_26_H_33_NO_7_Na [M + Na]^+^: 494.2149; Found: 494.2147. Based on the above analysis, the structure of hybrid **6a** was correct. 

The antiproliferative activity and cytotoxicity of DHA-5-methylisatin hybrids **6a–c** and **7a–m** against MCF-7, MDA-MB-231, MCF-7/ADR, and MDA-MB-231/ADR (Purchased from Procell) breast cancer cell lines as well as MCF-10A were assessed by 3-(4,5-dimethylthiazol-2-yl)-2,5-diphenyltetrazolium bromide (MTT) assay. The antiproliferative activity and cytotoxicity were expressed in terms of half maximal inhibitory concentration (IC_50_) values in Table 1 and Table 2, respectively.

As can be seen from Table 1, most of the synthesized hybrids (IC_50_: 15.3–99.9 µM) were active against MCF-7, MDA-MB-231, MCF-7/ADR, and MDA-MB-231/ADR breast cancer cell lines. The SARs illustrated that the carbon spacers between DHA and isatin moieties were the main parameter influenced the activity, and the relative contribution order was two-carbon > four-carbon > three-carbon. Hydrogen bond donors at C-3 position of isatin moiety could enhance the activity, and the relative contribution order was NOH > NNHCSNH_2_ > NNHCONH_2_. Additionally, alkyloxime at C-3 position of isatin moiety could improve the activity to some extent, while benzoxime decreased the activity.

From Table 2, all hybrids (IC_50_: >100 µM) displayed non-cytotoxicity towards normal MCF-10A breast cells, while the reference adriamycin (IC_50_: 68.8 µM) showed moderate cytotoxicity. The selectivity index (SI: IC_50(MCF-10A)_/IC_50(MCF-7)_ and IC_50(MCF-10A)_/IC_50(MDA-MB-231)_) values of seven hybrids were >2.07, and the resistance index (RI: IC_50(MCF-7/ADR)_/IC_50(MCF-7)_ and IC_50(MDA-MB-231/ADR)_/IC_50(MDA-MB-231)_) values were in a range of 0.78 to 1.22, demonstrating that the synthesized hybrids had no cross resistance with adriamycin.

Hybrid **7a** (IC_50_: 15.3–20.1 µM) was found to be most active against MCF-7, MDA-MB-231, MCF-7/ADR, and MDA-MB-231/ADR breast cancer cell lines. The activity was comparable to that of adriamycin (IC_50_: 18.9 µM) against MCF-7 cells, but >4.97 times superior to adriamycin (IC_50_: >100 µM) against MCF-7/ADR and MDA-MB-231/ADR cancer cell lines. The RI values of hybrid **7a** were 1.03 and 1.23 respectively, proving its potential to overcome drug resistance. Moreover, hybrid **7a** (IC_50_: >100 µM) was non-toxic towards normal MCF-10A breast cells, and the SI values were >5.15 and >6.53, revealing its excellent safety and selectivity profiles. It seems that the hydrogen bond donors especially NOH at C-3 position of isatin moiety showed higher SI values.

## 3. Experimental Section

### 3.1. Chemistry

#### 3.1.1. General

^1^H NMR and ^13^C spectra were determined on a Mercury-400 spectrometer (Varian, Palo Alto, CA, USA) in DMSO-*d*_6_ or CDCl_3_ using tetramethylsilane (TMS) as an internal standard. Electrospray ionization (ESI) mass spectra and high-resolution mass spectra (HRMS) were obtained on a Q-Tap mass spectrometer (MDSSCIEX, Concrod, CA, USA) and AccuTOF CS JMS-T100CS mass spectrometer (JEOL, Tokyo, Japan), respectively. Unless otherwise noted, the reagents were obtained from commercial supplier and used without further purification. 

#### 3.1.2. General Procedure for the Synthesis

To the mixture of dihydroartemisinin 1 (80 mmol) and ethylene glycol/1,3-propylene glycol/1,4-butanediol 2 (100 mmol) in DCM (500 mL), boron trifluoride diethyl etherate (20 mL) was added at 0 °C. The mixture was stirred at 25 °C for 12 h, and then quenched by sat. Na_2_CO_3_ (500 mL) slowly. The organic layer was washed with H_2_O (300 mL), dried over anhydrous Na_2_SO_4_, filtrated, and concentrated under reduced pressure. The residue was purified by silica gel chromatography eluted with PE to PE:EA = 1:1 to give intermediates **3a–c**. To the mixture of DHA intermediates **3a–c** (50 mmol) and triethylamine (100 mmol) in DCM (400 mL), *p*-toluenesulfonyl chloride (60 mmol) in was added. The mixture was stirred at room temperature for 5 h, and then H_2_O (300 mL) was added. The organic layer was dried over anhydrous Na_2_SO_4_, filtrated, and concentrated in vacuo to give crude toslyates **4a–c** which were used directly in the next step. The mixture of 5-methylisatin 5 (12 mmol) and potassium carbonate (K_2_CO_3_, 20 mmol) in DMF (50 mL) was stirred at room temperature for 30 min, and then crude toslyates **4a–c** (10 mmol) was added. The mixture was stirred overnight at room temperature, and then filtered. The filtrate was concentrated under reduced pressure to give the residue which was purified by silica gel chromatography eluted with PE to PE:EA = 1:1 to provide DHA-isatin hybrids **6a–c**. To a solution of hybrids **6a–c** (1 mmol) and hydroxyamine/methoxyamine/ethoxyamine/benzyloxyamine hydrochlorides or semicarbazide/thiosemicarbazide in a mixture of THF (10 mL) and H2O (10 mL), sodium acetate (2.0 mmol) was added. The mixture was stirred at 60 °C for 12 h, and after cooling to room temperature, the mixture was extracted with DCM (20 mL × 3). The combined organic layers were washed with H_2_O (30 mL), dried over anhydrous Na_2_SO_4_, filtrated, and concentrated under reduced pressure. The residue was purified by silica gel chromatography eluted with PE to PE:EA = 1:1 to give C-3 modified hybrids **7a–m**.

To the mixture of dihydroartemisinin 1 (80 mmol) and ethylene glycol/1,3-propylene glycol/1,4-butanediol 2 (100 mmol) in DCM (500 mL), boron trifluoride diethyl etherate (20 mL) was added at 0 °C. The mixture was stirred at 25 °C for 12 h, and then quenched by sat. Na_2_CO_3_ (500 mL) slowly. The organic layer was washed with H_2_O (300 mL), dried over anhydrous Na_2_SO_4_, filtrated, and concentrated under reduced pressure. The residue was purified by silica gel chromatography eluted with PE to PE:EA = 1:1 to give intermediates **3a–c**. To the mixture of DHA intermediates **3a–c** (50 mmol) and triethylamine (100 mmol) in DCM (400 mL), *p*-toluenesulfonyl chloride (60 mmol) in was added. The mixture was stirred at room temperature for 5 h, and then H_2_O (300 mL) was added. The organic layer was dried over anhydrous Na_2_SO_4_, filtrated, and concentrated in vacuo to give crude toslyates **4a–c** which were used directly in the next step. The mixture of 5-methylisatin 5 (12 mmol) and potassium carbonate (K_2_CO_3_, 20 mmol) in DMF (50 mL) was stirred at room temperature for 30 min, and then crude toslyates **4a–c** (10 mmol) was added. The mixture was stirred overnight at room temperature, and then filtered. The filtrate was concentrated under reduced pressure to give the residue which was purified by silica gel chromatography eluted with PE to PE:EA = 1:1 to provide DHA-isatin hybrids **6a–c**. To a solution of hybrids **6a–c** (1 mmol) and hydroxyamine/methoxyamine/ethoxyamine/benzyloxyamine hydrochlorides or semicarbazide/thiosemicarbazide in a mixture of THF (10 mL) and H2O (10 mL), sodium acetate (2.0 mmol) was added. The mixture was stirred at 60 °C for 12 h, and after cooling to room temperature, the mixture was extracted with DCM (20 mL × 3). The combined organic layers were washed with H_2_O (30 mL), dried over anhydrous Na_2_SO_4_, filtrated, and concentrated under reduced pressure. The residue was purified by silica gel chromatography eluted with PE to PE:EA = 1:1 to give C-3 modified hybrids **7a–m**.

##### 5-Methyl-1-(2-(((3R,5aS,6R,8aS,9R,12R,12aR)-3,6,9-trimethyldecahydro-12*H*-3,12-epoxy[1,2]dioxepino[4,3-i]isochromen-10-yl)oxy)ethyl)indoline-2,3-dione (**6a**)

Red solid. ^1^H NMR (600 Hz, CDCl_3_) 0.75–0.91 (m, 7H), 1.03–1.05 (m, 1H), 1.15–1.19 (m, 1H), 1.36–1.43 (m, 5H), 1.46–1.57 (m, 3H), 1.81–1.86 (m, 1H), 1.98–2.02 (m, 1H), 2.30–2.35 (m, 4H), 2.55–2.58 (m, 1H), 3.63–3.67 (m, 1H), 3.87–3.90 (m, 1H), 4.00–4.04 (m, 1H), 4.14–4.18 (m, 1H), 4.76 (d, *J* = 2.0 Hz, 1H), 5.19 (s, 1H), 6.86 (d, *J* = 4.0 Hz, 1H), 7.38 (d, *J* = 4.0 Hz, 1H), 7.41 (s, 1H). ^13^C NMR (150 Hz, CDCl_3_) 183.46, 158.46, 138.52, 133.52, 125.65, 117.58, 110.49, 104.14, 102.42, 87.80, 80.85, 64.60, 60.41, 52.33, 44.10, 40.03, 37.39, 36.32, 34.40, 30.66, 26.08, 24.62, 24.46, 20.64, 20.24, 12.81. HRMS-ESI: *m*/*z* Calcd for C_26_H_33_NO_7_Na [M + Na]^+^: 494.2149; Found: 494.2147.

##### 5-Methyl-1-(3-(((3R,5aS,6R,8aS,9R,12R,12aR)-3,6,9-trimethyldecahydro-12*H*-3,12-epoxy[1,2]dioxepino[4,3-i]isochromen-10-yl)oxy)propyl)indoline-2,3-dione (**6b**)

Brown solid. ^1^H NMR (600 Hz, DMSO-*d*_6_) δ 0.92–1.02 (m, 7H), 1.25–1.37 (m, 3H), 1.41–1.59 (m, 4H), 1.63–1.70 (m, 1H), 1.77–1.82 (m, 1H), 1.87–1.99 (m, 3H), 2.02–2.06 (m, 2H), 2.34 (s, 3H, CH3), 2.36–2.44 (m, 1H), 2.66–2.68 (m, 1H), 3.46–3.51 (m, 1H), 3.66–4.14 (m, 3H), 4.80 (d, *J* = 2.0 Hz, 1H), 5.54 (s, 1H), 6.82 (d, *J* = 4.0 Hz, 1H), 7.36–7.42 (m, 2H). ^13^C NMR (150 Hz, DMSO-*d*_6_) 183.98, 183.74, 158.48, 158.25, 148.79, 138.99, 138.80, 138.63, 133.53, 133.38, 125.92, 125.85, 125.63, 117.68, 117.57, 110.49, 110.00, 109.82, 104.29, 104.16, 102.14, 100.32, 91.23, 87.95, 81.03, 80.41, 66.06, 65.51, 58.86, 52.55, 51.66, 45.37, 44.38, 37.44, 36.40, 27.75, 26.16, 24.66, 24.57, 20.66, 20.65, 20.37, 14.20, 13.09, 12.72. HRMS-ESI: *m*/*z* Calcd for C_27_H_35_NO_7_Na [M + Na]^+^: 508.2306; Found: 508.2277.

##### 5-Methyl-1-(4-(((3R,5aS,6R,8aS,9R,12R,12aR)-3,6,9-trimethyldecahydro-12*H*-3,12-epoxy[1,2]dioxepino[4,3-i]isochromen-10-yl)oxy)butyl)indoline-2,3-dione (**6c**)

Yellow solid. ^1^H NMR (600 Hz, CDCl_3_) 0.85–0.96 (m, 7H), 1.22–1.32 (m, 3H), 1.40–1.53 (m, 5H), 1.68–1.79 (m, 7H), 1.85–1.89 (m, 1H), 2.01–2.05 (m, 1H), 2.33–2.40 (m, 4H), 2.60–2.63 (m, 1H), 3.39–3.49 (m, 1H), 3.71–4.02 (m, 3H), 4.76 (d, *J* = 2.0 Hz, 1H), 5.35 (s, 1H), 6.78 (d, *J* = 8.0 Hz, 1H), 7.37–7.42 (m, 2H). ^13^C NMR (150 Hz, CDCl_3_) 183.81, 158.27, 148.76, 138.93, 138.62, 133.50, 125.86, 117.69, 110.36, 109.88, 104.26, 104.12, 102.13, 100.02, 91.22, 87.92, 81.07, 67.77, 52.55, 51.68, 44.39, 40.05, 37.45, 36.43, 34.59, 30.88, 27.23, 26.18, 24.64, 24.51, 24.31, 20.66, 20.36, 13.04, 12.65. HRMS-ESI: *m*/*z* Calcd for C_28_H_37_NO_7_Na [M + Na]^+^: 522.2462; Found: 522.2461.

##### 3-(Hydroxyimino)-5-methyl-1-(2-(((3R,5aS,6R,8aS,9R,12R,12aR)-3,6,9-trimethyldecahydro-12*H*-3,12-epoxy[1,2]dioxepino[4,3-i]isochromen-10-yl)oxy)ethyl)indolin-2-one (**7a**)

Yellow solid. ^1^H NMR (600 Hz, CDCl_3_) 0.74–0.88 (m, 7H), 0.95–0.98 (m, 1H), 1.10–1.15 (m, 1H), 1.33–1.36 (m, 2H), 1.40 (s, 3H), 1.43–1.46 (m, 2H), 1.53–1.56 (m, 1H), 1.77–1.81 (m, 1H), 1.96–1.99 (m, 1H), 2.25–2.31 (m, 1H), 2.34 (s, 3H), 2.53–2.56 (m, 1H), 3.61–3.64 (m, 1H), 3.86–3.90 (m, 1H), 4.08–4.12 (m, 1H), 4.19–4.23 (m, 1H), 4.76 (d, *J* = 2.0 Hz, 1H), 5.12 (s, 1H), 6.82 (d, *J* = 4.0 Hz, 1H), 7.18 (d, *J* = 4.0 Hz, 1H), 7.95 (s, 1H), 10.98 (brs, 1H). ^13^C NMR (150 Hz, CDCl_3_) 144.51, 141.42, 132.72, 132.45, 128.80, 108.98, 104.09, 102.19, 87.77, 80.92, 64.47, 52.35, 44.16, 39.74, 37.50, 37.27, 36.35, 34.44, 30.70, 26.10, 24.59, 24.43, 20.96, 20.25, 12.80. HRMS-ESI: *m*/*z* Calcd for C_26_H_34_N_2_O_8_Na [M + Na]^+^: 509.2258; Found: 509.2266.

##### 3-(Hydroxyimino)-5-methyl-1-(3-(((3R,5aS,6R,8aS,9R,12R,12aR)-3,6,9-trimethyldecahydro-12*H*-3,12-epoxy[1,2]dioxepino[4,3-i]isochromen-10-yl)oxy)propyl)indolin-2-one (**7b**)

Yellow solid. ^1^H NMR (600 Hz, DMSO-*d*_6_) δ 0.84–0.99 (m, 7H), 1.24–1.37 (m, 3H), 1.43–1.59 (m, 5H), 1.64–1.68 (m, 1H), 1.78–1.82 (m, 1H), 1.87–1.98 (m, 2H), 2.01–2.06 (m, 2H), 2.34 (s, 3H, CH3), 2.36–2.44 (m, 1H), 2.64–2.68 (m, 1H), 3.43–3.52 (m, 1H), 3.70–4.14 (m, 3H), 4.81 (d, *J* = 2.0 Hz, 1H), 5.45 (s, 1H), 6.76 (d, *J* = 4.0 Hz, 1H), 7.16 (d, *J* = 4.0 Hz, 1H), 7.95 (s, 1H). ^13^C NMR (150 Hz, DMSO-*d*_6_) 164.25, 144.55, 141.38, 134.99, 132.77, 132.63, 132.49, 128.96, 128.79, 115.78, 108.84, 108.47, 108.30, 104.32, 104.19, 102.13, 100.30, 91.24, 89.70, 97.97, 81.11, 80.43, 66.29, 65.66, 60.45, 58.65, 52.57, 51.67, 51.44, 45.39, 44.44, 37.48, 37.41, 36.42, 34.66, 30.90, 28.01, 26.16, 24.70, 24.66, 24.57, 20.97, 20.39, 20.29, 13.09, 12.73. HRMS-ESI: *m*/*z* Calcd for C_27_H_36_N_2_O_7_Na [M + Na]^+^: 523.2415; Found: 523.2394.

##### 3-(Hydroxyimino)-5-methyl-1-(4-(((3R,5aS,6R,8aS,9R,12R,12aR)-3,6,9-trimethyldecahydro-12*H*-3,12-epoxy[1,2]dioxepino[4,3-i]isochromen-10-yl)oxy)butyl)indolin-2-one (**7c**)

Yellow solid. ^1^H NMR (600 Hz, CDCl_3_) 0.84–0.99 (m, 7H), 1.21–1.30 (m, 3H), 1.42–1.53 (m, 5H), 1.58–1.82 (m, 6H), 1.85–1.88 (m, 1H), 2.01–2.05 (m, 1H), 2.31–2.38 (m, 4H), 2.60–2.62 (m, 1H), 3.38–3.41 (m, 1H), 3.73–3.89 (m, 3H), 4.76 (d, *J* = 2.0 Hz, 1H), 5.36 (s, 1H), 6.14 (d, *J* = 4.0 Hz, 1H), 7.18 (d, *J* = 4.0 Hz, 1H), 7.93 (d, *J* = 4.0 Hz, 1H), 10.84 (brs, 1H). ^13^C NMR (150 Hz, CDCl_3_) 132.73, 108.39, 104.12, 102.08, 100.05, 91.22, 87.92, 81.11, 67.84, 52.57, 44.42, 39.80, 37.43, 36.43, 34.58, 30.90, 27.20, 26.18, 24.64, 24.50, 24.11, 20.97, 20.36, 20.28, 13.04, 12.64. HRMS-ESI: *m*/*z* Calcd for C_28_H_38_N_2_O_8_Na [M + Na]^+^: 537.2571; Found: 537.2568.

##### 2-(5-Methyl-2-oxo-1-(2-(((3R,5aS,6R,8aS,9R,12R,12aR)-3,6,9-trimethyldecahydro-12*H*-3,12-epoxy[1,2]dioxepino[4,3-i]isochromen-10-yl)oxy)ethyl)indolin-3-ylidene)hydrazine-1-carboxamide (**7d**)

Yellow solid. ^1^H NMR (600 Hz, CDCl_3_) 0.75–0.91 (m, 7H), 1.04–1.06 (m, 1H), 1.14–1.18 (m, 1H), 1.36–1.40 (m, 5H), 1.46–1.51 (m, 2H), 1.56–1.59 (m, 1H), 1.81–1.85 (m, 1H), 1.98–2.01 (m, 1H), 2.30–2.35 (m, 4H), 2.55–2.58 (m, 1H), 3.63–3.67 (m, 1H), 3.86–3.90 (m, 1H), 4.00–4.03 (m, 1H), 4.14–4.18 (m, 1H), 4.76 (d, *J* = 2.0 Hz, 1H), 5.19 (s, 1H), 6.86 (d, *J* = 4.0 Hz, 1H), 7.38 (d, *J* = 4.0 Hz, 1H), 7.41 (s, 1H). ^13^C NMR (150 Hz, CDCl_3_) 183.47, 158.47, 148.82, 138.52, 133.52, 125.65, 117.58, 110.49, 104.15, 102.42, 87.81, 80.85, 64.61, 52.34, 44.10, 40.04, 37.39, 36.32, 34.40, 30.66, 26.09, 24.62, 24.46, 20.65, 20.25, 12.82.

##### 2-(5-Methyl-2-oxo-1-(4-(((3R,5aS,6R,8aS,9R,12R,12aR)-3,6,9-trimethyldecahydro-12*H*-3,12-epoxy[1,2]dioxepino[4,3-i]isochromen-10-yl)oxy)butyl)indolin-3-ylidene)hydrazine-1-carboxamide (**7e**)

Yellow solid. ^1^H NMR (600 Hz, CD_3_OD) 0.72–0.87 (m, 7H), 1.06–1.14 (m, 2H), 1.22–1.32 (m, 5H), 1.39–1.48 (m, 1H), 1.55–1.77 (m, 7H), 1.90–1.94 (m, 1H), 2.16–2.25 (m, 4H), 2.39–2.42 (m, 1H), 3.36–3.86 (m, 4H), 4.60 (d, *J* = 2.0 Hz, 1H), 5.23 (s, 1H), 6.92 (d, *J* = 4.0 Hz, 1H), 7.30–7.39 (m, 2H). ^13^C NMR (150 Hz, DMSO-*d*_6_) 187.55, 162.82, 152.73, 142.47, 142.33, 137.44, 128.74, 121.78, 114.21, 107.96, 106.25, 104.00, 95.17, 91.78, 84.71, 84.22, 71.85, 56.47, 48.36, 43.48, 41.17, 39.90, 38.28, 34.92, 28.56, 28.27, 28.24, 28.19, 23.30, 23.14, 15.90, 15.52.

##### 2-(5-Methyl-2-oxo-1-(3-(((3R,5aS,6R,8aS,9R,12R,12aR)-3,6,9-trimethyldecahydro-12*H*-3,12-epoxy[1,2]dioxepino[4,3-i]isochromen-10-yl)oxy)propyl)indolin-3-ylidene)hydrazine-1-carbothioamide (**7f**)

Yellow solid. ^1^H NMR (600 Hz, CD_3_OD) 0.93–1.02 (m, 7H), 1.23–1.34 (m, 2H), 1.40–1.50 (m, 4H), 1.52–1.59 (m, 1H), 1.65–1.73 (m, 2H), 1.77–1.91 (m, 2H), 1.94–2.06 (m, 3H), 2.34–2.39 (m, 4H), 2.64–2.66 (m, 3H), 3.42–3.49 (m, 1H), 3.73–4.05 (m, 3H), 4.80 (d, *J* = 2.0 Hz, 1H), 5.48 (s, 1H), 6.62 (brs, 1H), 6.80 (d, *J* = 4.0 Hz, 1H), 7.18 (d, *J* = 4.0 Hz, 1H), 7.40 (s, 1H), 7.52 (brs, 1H), 12.88 (s, 1H). ^13^C NMR (150 Hz, DMSO-*d*_6_) 180.01, 161.11, 141.13, 132.91, 132.00, 121.57, 119.44, 109.12, 104.17, 102.17, 87.96, 81.12, 65.34, 52.66, 44.42, 37.35, 37.22, 36.43, 30.90, 27.90, 26.18, 24.69, 24.54, 21.02, 20.47, 13.09, 12.71. HRMS-ESI: *m*/*z* Calcd for C_28_H_38_N_4_O_6_SNa [M + Na]^+^: 581.2404; Found: 581.2377.HRMS-ESI: *m*/*z* Calcd for C_28_H_37_N_4_O_6_S [M − H]^+^: 577.2439; Found: 577.2428.

##### 2-(5-Methyl-2-oxo-1-(4-(((3R,5aS,6R,8aS,9R,12R,12aR)-3,6,9-trimethyldecahydro-12*H*-3,12-epoxy[1,2]dioxepino[4,3-i]isochromen-10-yl)oxy)butyl)indolin-3-ylidene)hydrazine-1-carbothioamide (**7g**)

Yellow solid. ^1^H NMR (600 Hz, DMSO-*d*_6_) 0.76–0.89 (m, 7H), 1.08–1.18 (m, 2H), 1.23–1.34 (m, 5H), 1.40–1.49 (m, 1H), 1.54–1.70 (m, 6H), 1.72–1.77 (m, 1H), 1.95–1.98 (m, 1H), 2.12–2.17 (m, 1H), 2.34–2.36 (m, 1H), 3.36–3.44 (m, 1H), 3.67–3.78 (m, 3H), 4.65 (d, *J* = 2.0 Hz, 1H), 5.21 (s, 1H), 7.06 (d, *J* = d.0 Hz, 1H), 7.22 (d, *J* = 4.0 Hz, 1H), 7.55 (s, 1H), 8.69 (s, 1H), 9.03 (s, 1H), 12.40 (s, 1H). ^13^C NMR (150 Hz, DMSO-*d*_6_) 183.93, 165.98, 145.97, 137.12, 126.63, 126.41, 126.49, 124.69, 114.93, 108.49, 106.32, 92.15, 85.64, 72.50, 57.27, 49.03, 41.91, 35.73, 30.78, 25.83, 25.44, 17.98. HRMS-ESI: *m*/*z* Calcd for C_29_H_40_N_4_O_6_SNa [M + Na]^+^: 595.2561; Found: 595.2525.

##### 3-(Methoxyimino)-1-(4-(((3R,5aS,6R,8aS,9R,12R,12aR)-3,6,9-trimethyldecahydro-12*H*-3,12-epoxy[1,2]dioxepino[4,3-i]isochromen-10-yl)oxy)butyl)indolin-2-one (**7h**)

Yellow solid. ^1^H NMR (600 Hz, CDCl_3_) 0.85–0.96 (m, 7H), 1.20–1.30 (m, 2H), 1.41–1.52 (m, 5H), 1.57–1.78 (m, 7H), 1.84–1.89 (m, 1H), 2.01–2.04 (m, 1H), 2.33–2.38 (m, 1H), 2.59–2.63 (m, 1H), 3.37–3.41 (m, 1H), 3.73–3.82 (m, 2H), 3.85–3.88 (m, 1H), 4.30 (s, 3H), 4.76 (d, *J* = 2.0 Hz, 1H), 5.35 (s, 1H), 6.82 (d, *J* = 8.0 Hz, 1H), 7.06 (t, *J* = 8.0 Hz, 1H), 7.38 (t, *J* = 4.0 Hz, 1H), 7.96 (d, *J* = 4.0 Hz, 1H),. ^13^C NMR (150 Hz, CDCl_3_) 163.47, 143.85, 143.61, 132.38, 128.04, 122.82, 115.88, 108.58, 104.08, 102.04, 87.90, 81.10, 67.80, 64.75, 52.57, 44.42, 39.73, 37.43, 36.44, 34.59, 30.89, 27.17, 26.20, 24.65, 24.49, 24.47, 20.35, 13.03. HRMS-ESI: *m*/*z* Calcd for C_28_H_38_N_2_O_7_Na [M + Na]^+^: 537.2555; Found: 537.2571.

##### 3-(Ethoxyimino)-5-methyl-1-(2-(((3R,5aS,6R,8aS,9R,12R,12aR)-3,6,9-trimethyldecahydro-12*H*-3,12-epoxy[1,2]dioxepino[4,3-i]isochromen-10-yl)oxy)ethyl)indolin-2-one (**7i**)

Yellow solid. ^1^H NMR (600 Hz, CDCl_3_) 0.73–0.90 (m, 7H), 0.92–1.00 (m, 1H), 1.33–1.36 (m, 2H), 1.40 (s, 3H), 1.43–1.47 (m, 5H), 1.54–1.57 (m, 1H), 1.79–1.82 (m, 1H), 1.96–2.02 (m, 1H), 2.29–2.32 (m, 1H), 2.34 (s, 3H), 2.52–2.55 (m, 1H), 3.59–3.62 (m, 1H), 3.83–3.87 (m, 1H), 4.07–4.12 (m, 1H), 4.16–4.20 (m, 1H), 4.56 (q, *J* = 8.0 Hz, 1H), 4.74 (d, *J* = 4.0 Hz, 1H), 5.12 (s, 1H), 6.80 (d, *J* = 8.0 Hz, 1H), 7.16 (d, *J* = 8.0 Hz, 1H), 7.78 (s, 1H). ^13^C NMR (150 Hz, CDCl_3_) 163.79, 143.53, 141.54, 132.37, 132.30, 128.38, 115.85, 108.88, 104.03, 102.18, 87.73, 80.90, 72.94, 64.52, 52.36, 44.18, 39.59, 37.26, 36.36, 34.47, 30.71, 26.11, 24.60, 24.42, 21.00, 20.25, 14.80, 12.80. HRMS-ESI: *m*/*z* Calcd for C_27_H_39_N_2_O_7_ [M + H_3_O]^+^: 519.2701; Found: 519.2194.

##### 3-(Ethoxyimino)-5-methoxy-1-(4-(((3R,5aS,6R,8aS,9R,12R,12aR)-3,6,9-trimethyldecahydro-12*H*-3,12-epoxy[1,2]dioxepino[4,3-i]isochromen-10-yl)oxy)butyl)indolin-2-one (**7j**)

Yellow solid. ^1^H NMR (600 Hz, CDCl_3_) 0.84–0.96 (m, 7H), 1.20–1.30 (m, 2H), 1.41–1.51 (m, 7H), 1.58–1.66 (m, 4H), 1.68–1.77 (m, 4H), 1.85–1.88 (m, 1H), 2.01–2.04 (m, 1H), 2.33–2.38 (m, 4H), 2.59–2.62 (m, 1H), 3.36–3.40 (m, 1H), 3.71–3.78 (m, 2H), 3.84–3.88 (m, 1H), 4.56 (q, *J* = 4.0 Hz, 1H), 4.76 (d, *J* = 4.0 Hz, 1H), 5.35 (s, 1H), 6.72 (d, *J* = 4.0 Hz, 1H), 7.17 (d, *J* = 4.0 Hz, 1H), 7.80 (s, 1H). ^13^C NMR (150 Hz, CDCl_3_) 163.63, 143.64, 141.52, 132.48, 132.30, 128.56, 108.28, 104.04, 102.04, 87.90, 81.11, 72.94, 67.85, 52.58, 44.43, 39.70, 37.43, 36.45, 34.59, 30.91, 27.17, 26.21, 24.65, 24.50, 21.01, 20.36, 14.80, 13.04. HRMS-ESI: *m*/*z* Calcd for C_30_H_42_N_2_O_7_Na [M + Na]^+^: 565.2884; Found: 565.2850.

##### 3-((Benzyloxy)imino)-5-methyl-1-(2-(((3R,5aS,6R,8aS,9R,12R,12aR)-3,6,9-trimethyldecahydro-12*H*-3,12-epoxy[1,2]dioxepino[4,3-i]isochromen-10-yl)oxy)ethyl)indolin-2-one (**7k**)

Yellow solid. ^1^H NMR (600 Hz, CDCl_3_) 0.74–0.88 (m, 7H), 0.95–0.99 (m, 1H), 1.11–1.16 (m, 1H), 1.31–1.36 (m, 2H), 1.40–1.50 (m, 5H), 1.52–1.57 (m, 1H), 1.78–1.82 (m, 1H), 1.97–2.00 (m, 1H), 2.29 (s, 3H), 2.31–2.34 (m, 1H), 2.52–2.55 (m, 1H), 3.59–3.62 (m, 1H), 3.83–3.87 (m, 1H), 4.06–4.09 (m, 1H), 4.14–4.18 (m, 1H), 4.74 (d, *J* = 2.0 Hz, 1H), 5.13 (s, 1H), 5.25 (s, 2H), 6.80 (d, *J* = 4.0 Hz, 1H), 7.16 (d, *J* = 4.0 Hz, 1H), 7.34–7.47 (m, 5H), 7.75 (s, 1H). ^13^C NMR (150 Hz, CDCl_3_) 163.67, 144.04, 141.67, 136.43, 132.60, 132.40, 128.61, 128.59, 128.42, 128.37, 128.32, 115.79, 108.93, 104.04, 102.22, 87.74, 80.91, 79.30, 64.55, 52.35, 44.18, 39.64, 37.27, 36.36, 34.45, 30.71, 26.12, 24.61, 24.42, 20.99, 20.24, 12.81. HRMS-ESI: *m*/*z* Calcd for C_33_H_40_N_2_O_7_Na [M + Na]^+^: 599.2728; Found: 599.2715.

##### 3-((Benzyloxy)imino)-6-methyl-1-(3-(((3R,5aS,6R,8aS,9R,12R,12aR)-3,6,9-trimethyldecahydro-12*H*-3,12-epoxy[1,2]dioxepino[4,3-i]isochromen-10-yl)oxy)propyl)indolin-2-one (**7l**)

This compound was obtained directly from the collaborators and the detailed synthesis method, together with the characterization data have been reported in Literature [21].

##### 3-((Benzyloxy)imino)-5-methyl-1-(4-(((3R,5aS,6R,8aS,9R,12R,12aR)-3,6,9-trimethyldecahydro-12*H*-3,12-epoxy[1,2]dioxepino[4,3-i]isochromen-10-yl)oxy)butyl)indolin-2-one (**7m**)

Yellow solid. ^1^H NMR (600 Hz, CDCl_3_) 0.84–0.98 (m, 7H), 1.22–1.30 (m, 3H), 1.41–1.52 (m, 5H), 1.57–1.77 (m, 6H), 1.85–1.88 (m, 1H), 2.00–2.04 (m, 1H), 2.29 (s, 3H), 2.33–2.38 (m, 1H), 2.59–2.61 (m, 1H), 3.36–3.40 (m, 1H), 3.69–3.88 (m, 3H), 4.76 (d, *J* = 2.0 Hz, 1H), 5.35 (s, 1H), 5.53 (s, 2H), 6.72 (d, *J* = 8.0 Hz, 1H), 7.16 (d, *J* = 8.0 Hz, 1H), 7.35–7.47 (m, 2H), 7.77 (s, 1H). ^13^C NMR (150 Hz, CDCl_3_) 163.50, 144.17, 141.65, 136.48, 132.73, 132.40, 128.80, 128.60, 128.40, 128.34, 128.30, 115.95, 108.33, 104.08, 102.05, 87.91, 81.11, 79.26, 67.84, 52.58, 51.69, 44.43, 39.75, 37.42, 36.45, 34.60, 30.91, 27.18, 26.21, 24.66, 24.49, 21.00, 20.36, 13.05. HRMS-ESI: *m*/*z* Calcd for C_35_H_44_N_2_O_8_Na [M + Na]^+^: 627.3041; Found: 627.3032.

### 3.2. Pharmacological/Biological Assays

MCF-7, MDA-MB-231, MCF-7/ADR, and MDA-MB-231/ADR breast cancer cell lines (2 × 10^3^) were plated in each well of a 96-well plate and were allowed to adhere and spread for 24 h. The dihydroartemisinin-5-methylisatin hybrids **6a–e** and **7a–m** were added to a final concentration of 100 µM, and the cells were cultured for 24 h at 37 °C. 3-(4,5-dimethyl-2-thiazolyl)-2,5-diphenyltetrazolium bromide (MTT) solution (10 µL) was added to each well, and the cultures were incubated for an additional 4 h. A further 100 µL of MTT solution was added and incubation continued overnight. The absorbance at 540 nm was determined in each well with a 96-well plate reader. The growth of the treated cells was compared with that of untreated cells.

## 4. Conclusions

In this study, the influence of the carbon spacers between DHA and isatin moieties in DHA-5-methylisatin hybrids were investigated. The preliminary results illustrated that the length of the carbon spacer was the main parameter affected the activity, and hybrids tethered via the two-carbon linker showed the highest activity. Hydrogen bond donors at C-3 position of isatin moiety could enhance the activity and alkyloxime at C-3 position of isatin moiety could improve the activity to some extent, while benzoxime decreased the activity. The enriched SARs may facilitate further structural modifications.

In particular, the representative hybrid **7a** not only demonstrated profound activity against both drug-sensitive and drug-resistant breast cancer cell lines, but also possessed excellent safety and selectivity profile. Therefore, hybrid **7a** was a promising candidate for the treatment of both drug-sensitive and drug-resistant breast cancers and worthy of further preclinical evaluations. 

## Figures and Tables

**Table 1:** The antiproliferative activity of DHA-5-methylisatin hybrids **6a–c** and **7a–m**.

Hybrid	Antiproliferative Activity (IC_50_: µM)			
	MCF-7	MDA-MB-231	MCF-7/ADR	MDA-MB-231/ADR
**6a**	31.7	29.1	25.3	26.9
**6b**	>100	>100	87.4	>100
**6c**	59.7	42.3	49.9	44.5
**7a**	19.4	15.3	20.1	18.8
**7b**	30.3	42.6	28.7	36.4
**7c**	22.1	26.7	23.4	28.5
**7d**	27.2	19.7	33.3	21.6
**7e**	39.0	31.2	42.1	34.5
**7r**	46.7	48.2	36.4	51.1
**7g**	28.9	35.1	30.3	40.6
**7h**	85.2	73.9	77.5	80.3
**7i**	69.3	70.4	61.8	72.7
**7j**	99.9	86.8	90.4	97.1
**7k**	>100	>100	>100	>100
**7l**	>100	>100	>100	>100
**7m**	>100	>100	>100	>100
**ART ^a^**	87.6	72.4	>100	>100
**DHA ^b^**	73.2	69.6	79.1	82.8
**ADR ^c^**	18.9	4.46	>100	>100

^a^ ART: Artemisinin;. ^b^ DHA: Dihydroartemisinin; ^c^ ADR: Adriamycin.

**Table 2:** The cytotoxicity, selectivity index and resistance index values of DHA-5-methylisatin hybrids **6a–c** and **7a–m**.

Hybrid	Cytotoxicity (IC_50_: µM)	SI ^a^		RI ^d^	
	MCF-10A	SI1 ^b^	SI2 ^c^	RI1 ^e^	RI2 ^f^
**6a**	>100	>3.15	>3.43	0.80	0.92
**6b**	>100	--	--	<0.87	--
**6c**	>100	>1.67	>2.36	0.83	1.05
**7a**	>100	>5.15	>6.53	1.03	1.23
**7b**	>100	>3.30	>2.34	0.95	0.85
**7c**	>100	>4.52	>3.83	1.06	1.07
**7d**	>100	>3.67	>5.07	1.22	1.10
**7e**	>100	>2.56	>3.20	1.08	1.11
**7f**	>100	>2.14	>2.07	0.78	1.06
**7g**	>100	>3.46	>2.84	1.05	1.16
**7h**	>100	>1.17	>1.35	0.91	1.09
**7i**	>100	>1.44	>1.42	0.89	1.03
**7j**	>100	>1.00	>1.15	0.90	1.12
**7k**	>100	--	--	--	--
**7l**	>100	--	--	--	--
**7m**	>100	--	--	--	--
**ART**	>100	>1.14	>1.38	>1.14	>1.38
**DHA**	>100	>1.36	>1.43	1.08	1.19
**ADR**	68.8	3.64	14.76	>5.29	>22.42

^a^ SI: Selectivity index, IC_50(normal cell)_/IC_50(cancer cell)_; ^b^ SI1: IC_50(MCF-10A)_/IC_50(MCF-7)_; ^c^ SI1: IC_50(MCF-10A)_/IC_50(MDA-MB-231)_; ^d^ RI: Resistance index, IC_50(drug-resistant cancer cell)_/IC_50(drug-sensitive cancer cell)_; ^e^ RI1: IC_50(MCF-7/ADR)_/IC_50(MCF-7)_; ^f^ RI2: IC_50(MDA-MB-231/ADR)_/IC_50(MDA-MB-231)_.

## Data Availability

Not applicable.

## Supplementary Materials

The following supporting information can be downloaded at: https://www.mdpi.com/article/10.3390/molecules27227994/s1, Figures: NMR analysis and MS analysis of the compounds.

## Sample Availability

Samples of the compounds are available from the authors.
