# Supplementary material for: The Anti-Breast Cancer Activity of Dihydroartemisinin-5-methylisatin Hybrids Tethered via Different Carbon Spacers
Source: Molecules. 2022 Nov 18;27(22):7994. doi: 10.3390/molecules27227994 (PMC9695041; doi:10.3390/molecules27227994)

The anti-breast cancer activity of dihydroartemisinin-5-methylisatin hybrids tethered *via* different carbon spacers

Yanfen Yao [1], Hong Wang [1], Jing Xu [1], Feng Gao [2], Wei Cao [3,4,*]

[1] Shandong Provincial Third Hospital, Shandong University, Jinan 250031, China
[2] State Key Laboratory of Biobased Material and Green Papermaking (LBMP), Qilu University of Technology, Jinan, 250353, China
[3] Department of Nephrology, The First Affiliated Hospital of Shandong First Medical University & Shandong Provincial Qianfoshan Hospital, Shandong Institute of Nephrology, Jinan, 250014, China
[4] The First Clinical College, Shandong University of Traditional Chinese Medicine, Jinan, 250355, China
* Correspondence: caowei@sdhospital.com.cn

5-methyl-1-(2-(((3R,5aS,6R,8aS,9R,12R,12aR)-3,6,9-trimethyldecahydro-12H-3,12-epoxy[1,2]dioxepino[4,3-i]isochromen-10-yl)oxy)ethyl)indoline-2,3-dione (**6a**)

Red solid. ^{1}H NMR (600 Hz, $CDCl_3$) 0.75–0.91 (m, 7H), 1.03–1.05 (m, 1H), 1.15–1.19 (m, 1H), 1.36–1.43 (m, 5H), 1.46–1.57 (m, 3H), 1.81–1.86 (m, 1H), 1.98–2.02 (m, 1H), 2.30–2.35 (m, 4H), 2.55–2.58 (m, 1H), 3.63–3.67 (m, 1H), 3.87–3.90 (m, 1H), 4.00–4.04 (m, 1H), 4.14–4.18 (m, 1H), 4.76 (d, J = 2.0 Hz, 1H), 5.19 (s, 1H), 6.86 (d, J = 4.0 Hz, 1H), 7.38 (d, J = 4.0 Hz, 1H), 7.41 (s, 1H). ^{13}C NMR (150 Hz, $CDCl_3$) 183.46, 158.46, 138.52, 133.52, 125.65, 117.58, 110.49, 104.14, 102.42, 87.80, 80.85, 64.60, 60.41, 52.33, 44.10, 40.03, 37.39, 36.32, 34.40, 30.66, 26.08, 24.62, 24.46, 20.64, 20.24, 12.81. HRMS-ESI: m/z Calcd for $C_{26}H_{33}NO_7Na$ $[M+Na]^+$: 494.2149; Found: 494.2147.

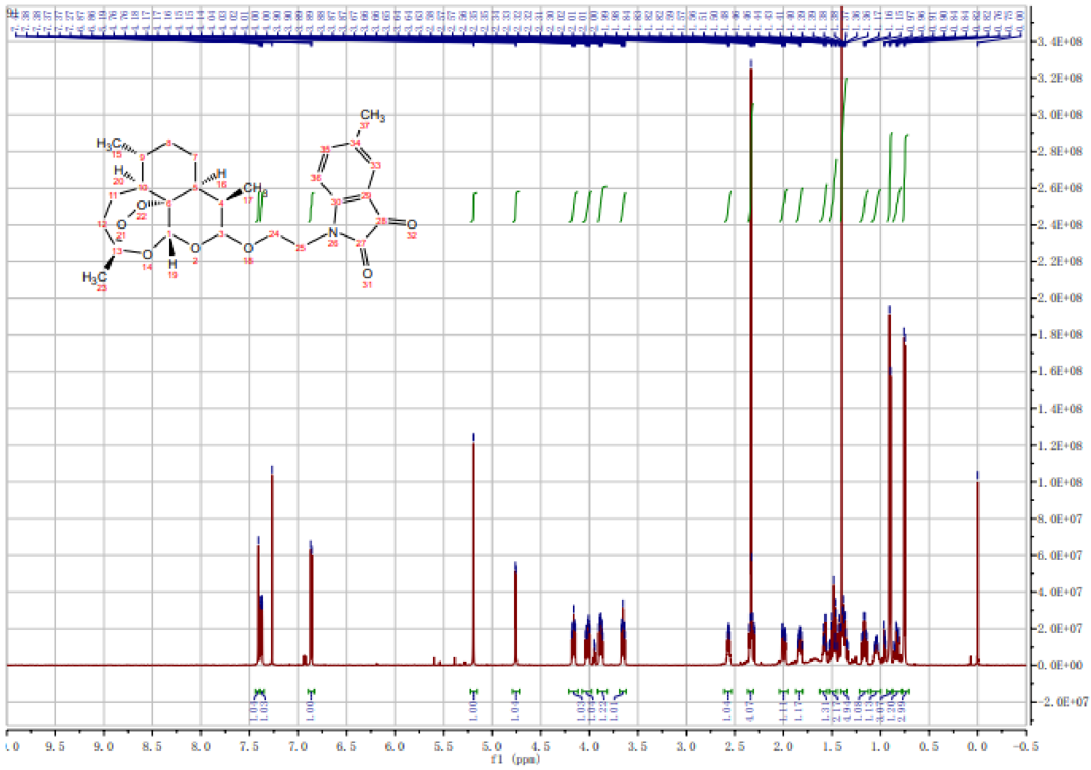

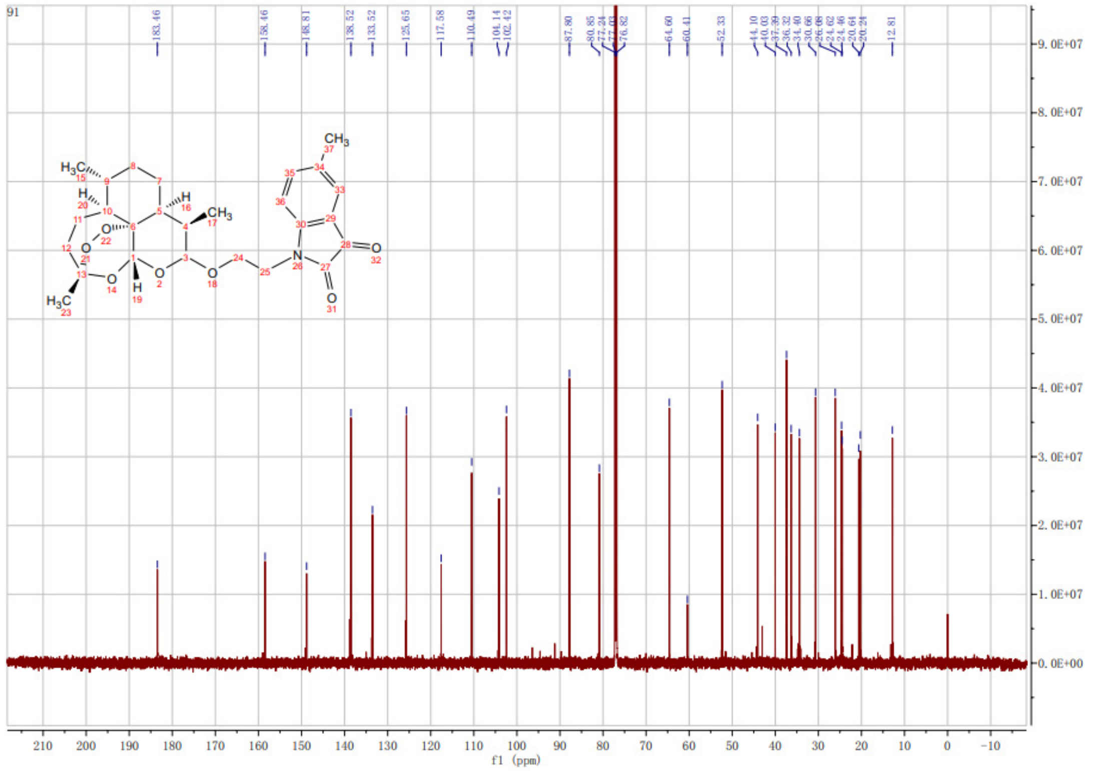

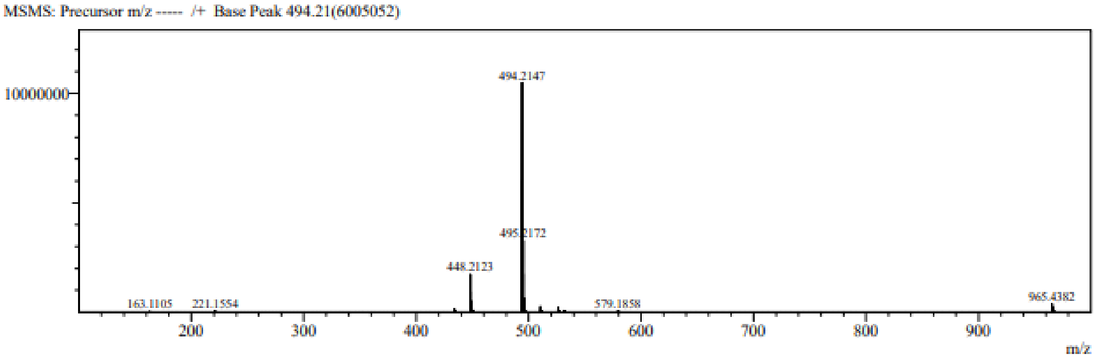

5-methyl-1-(3-(((3R,5aS,6R,8aS,9R,12R,12aR)-3,6,9-trimethyldecahydro-12H-3,12-epoxy[1,2]dioxepino[4,3-i]isochromen-10-yl)oxy)propyl)indoline-2,3-dione (**6b**)

Brown solid. ^{1}H NMR (600 Hz, DMSO-d_6) δ 0.92–1.02 (m, 7H), 1.25–1.37 (m, 3H), 1.41–1.59 (m, 4H), 1.63–1.70 (m, 1H), 1.77–1.82 (m, 1H), 1.87–1.99 (m, 3H), 2.02–2.06 (m, 2H), 2.34 (s, 3H, CH3), 2.36–2.44 (m, 1H), 2.66–2.68 (m, 1H), 3.46–3.51 (m, 1H), 3.66–4.14 (m, 3H), 4.80 (d, J = 2.0 Hz, 1H), 5.54 (s, 1H), 6.82 (d, J = 4.0 Hz, 1H), 7.36–7.42 (m, 2H). ^{13}C NMR (150 Hz, DMSO-d_6) 183.98, 183.74, 158.48, 158.25, 148.79, 138.99, 138.80, 138.63, 133.53, 133.38, 125.92, 125.85, 125.63, 117.68, 117.57, 110.49, 110.00, 109.82, 104.29, 104.16, 102.14, 100.32, 91.23, 87.95, 81.03, 80.41, 66.06, 65.51, 58.86, 52.55, 51.66, 45.37, 44.38, 37.44, 36.40, 27.75, 26.16, 24.66, 24.57, 20.66, 20.65, 20.37, 14.20, 13.09, 12.72. HRMS-ESI: m/z Calcd for $C_{27}H_{35}NO_7Na$ [M+Na]$^+$: 508.2306; Found: 508.2277.

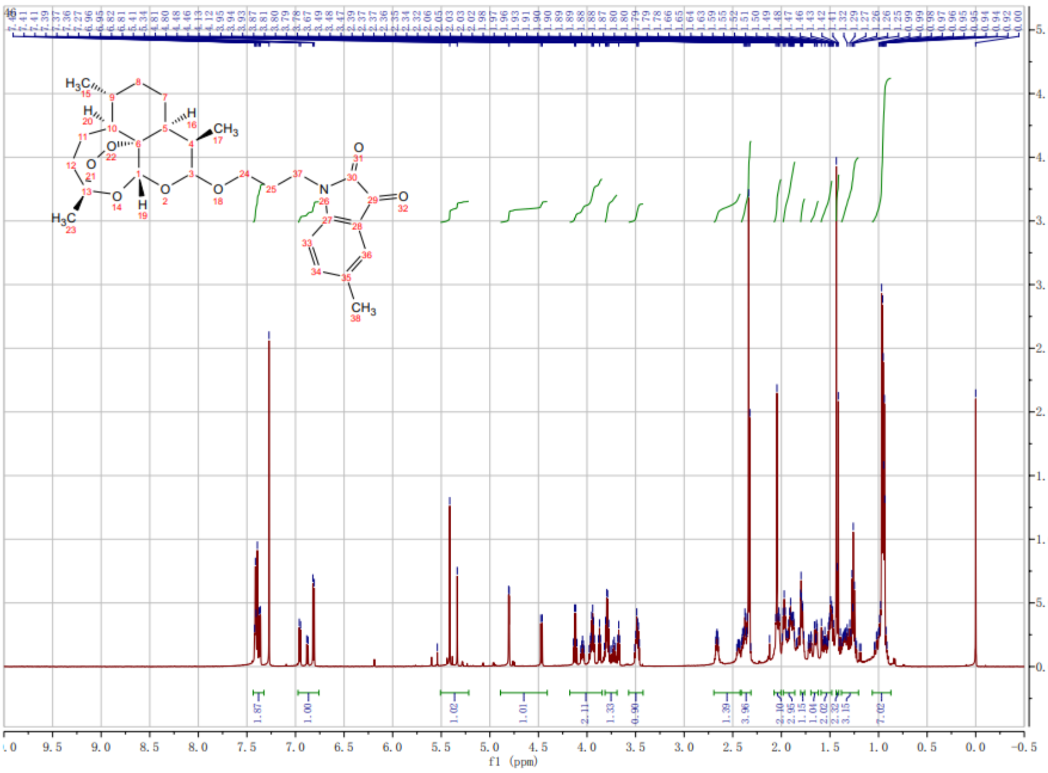

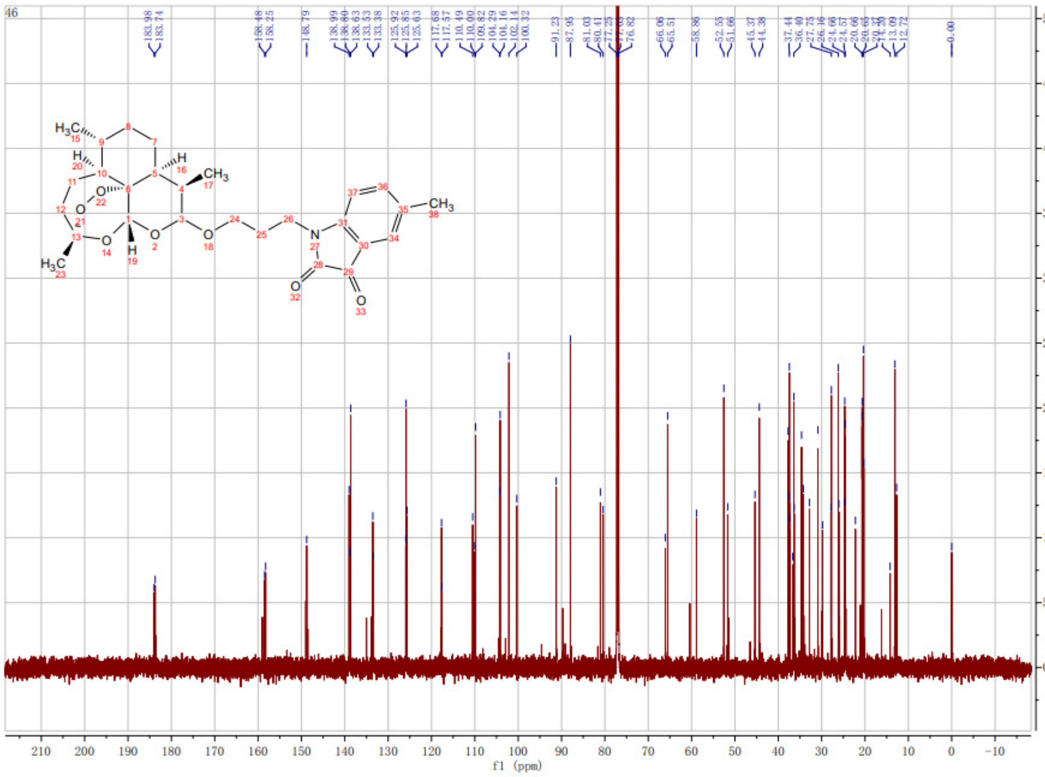

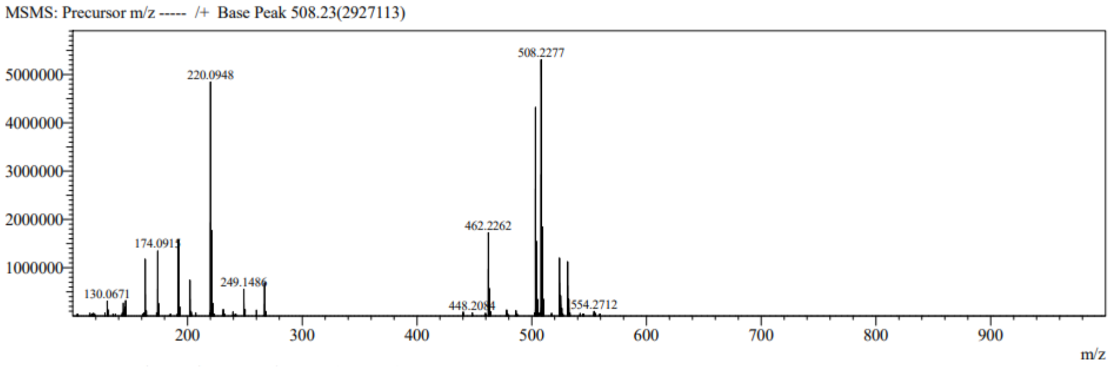

5-methyl-1-(4-(((3R,5aS,6R,8aS,9R,12R,12aR)-3,6,9-trimethyldecahydro-12H-3,12-epoxy[1,2]dioxepino[4,3-i]isochromen-10-yl)oxy)butyl)indoline-2,3-dione (**6c**)

Yellow solid. ^{1}H NMR (600 Hz, $CDCl_3$) 0.85–0.96 (m, 7H), 1.22–1.32 (m, 3H), 1.40–1.53 (m, 5H), 1.68–1.79 (m, 7H), 1.85–1.89 (m, 1H), 2.01–2.05 (m, 1H), 2.33–2.40 (m, 4H), 2.60–2.63 (m, 1H), 3.39–3.49 (m, 1H), 3.71–4.02 (m, 3H), 4.76 (d, J = 2.0 Hz, 1H), 5.35 (s, 1H), 6.78 (d, J = 8.0 Hz, 1H), 7.37–7.42 (m, 2H). ^{13}C NMR (150 Hz, $CDCl_3$) 183.81, 158.27, 148.76, 138.93, 138.62, 133.50, 125.86, 117.69, 110.36, 109.88, 104.26, 104.12, 102.13, 100.02, 91.22, 87.92, 81.07, 67.77, 52.55, 51.68, 44.39, 40.05, 37.45, 36.43, 34.59, 30.88, 27.23, 26.18, 24.64, 24.51, 24.31, 20.66, 20.36, 13.04, 12.65. HRMS-ESI: m/z Calcd for $C_{28}H_{37}NO_7Na$ $[M+Na]^+$: 522.2462; Found: 522.2461.

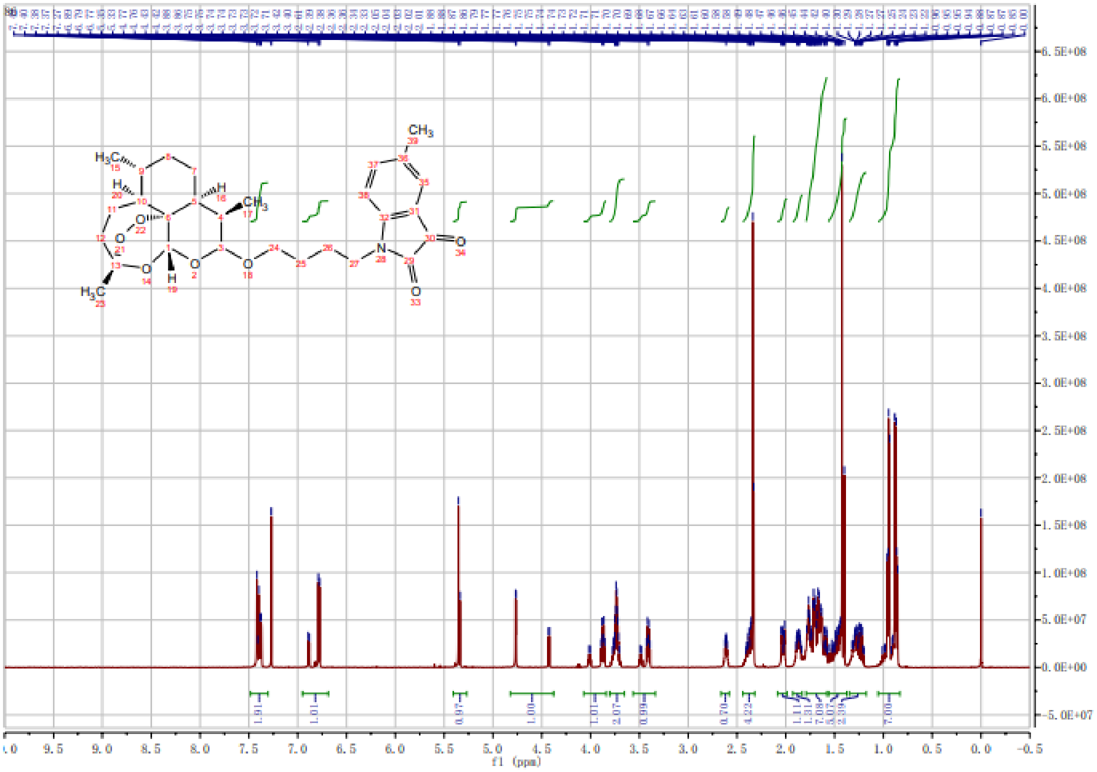

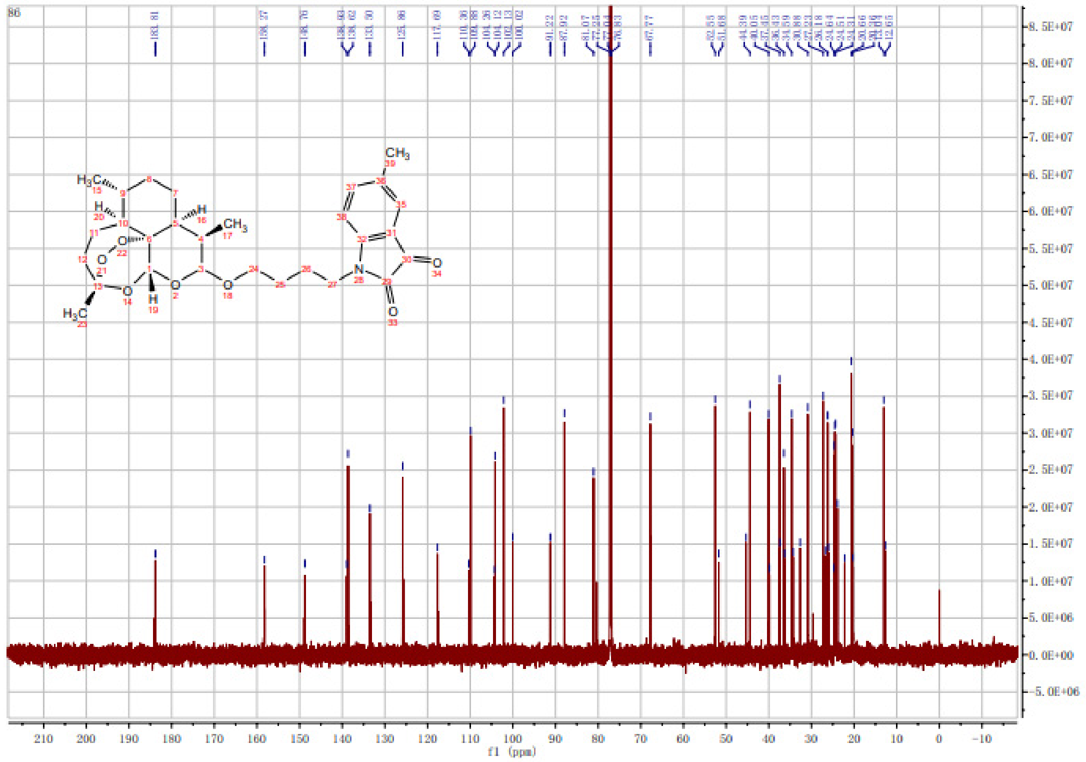

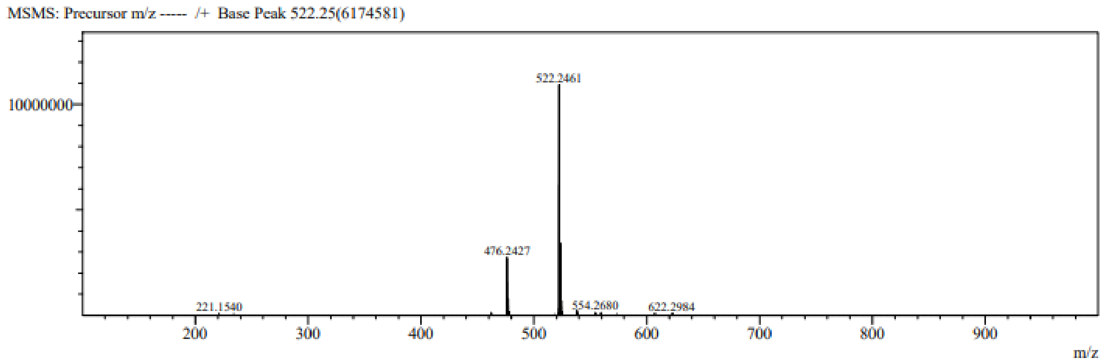

3-(hydroxyimino)-5-methyl-1-(2-(((3R,5aS,6R,8aS,9R,12R,12aR)-3,6,9-trimethyldecahydro-12H-3,12-epoxy[1,2]dioxepino[4,3-i]isochromen-10-yl)oxy)ethyl)indolin-2-one (**7a**)

Yellow solid. ^{1}H NMR (600 Hz, $CDCl_3$) 0.74–0.88 (m, 7H), 0.95–0.98 (m, 1H), 1.10–1.15 (m, 1H), 1.33–1.36 (m, 2H), 1.40 (s, 3H), 1.43–1.46 (m, 2H), 1.53–1.56 (m, 1H), 1.77–1.81 (m, 1H), 1.96–1.99 (m, 1H), 2.25–2.31 (m, 1H), 2.34 (s, 3H), 2.53–2.56 (m, 1H), 3.61–3.64 (m, 1H), 3.86–3.90 (m, 1H), 4.08–4.12 (m, 1H), 4.19–4.23 (m, 1H), 4.76 (d, J = 2.0 Hz, 1H), 5.12 (s, 1H), 6.82 (d, J = 4.0 Hz, 1H), 7.18 (d, J = 4.0 Hz, 1H), 7.95 (s, 1H), 10.98 (brs, 1H). ^{13}C NMR (150 Hz, $CDCl_3$) 144.51, 141.42, 132.72, 132.45, 128.80, 108.98, 104.09, 102.19, 87.77, 80.92, 64.47, 52.35, 44.16, 39.74, 37.50, 37.27, 36.35, 34.44, 30.70, 26.10, 24.59, 24.43, 20.96, 20.25, 12.80. HRMS-ESI: m/z Calcd for $C_{26}H_{34}N_2O_8Na$ $[M+Na]^+$: 509.2258; Found: 509.2266.

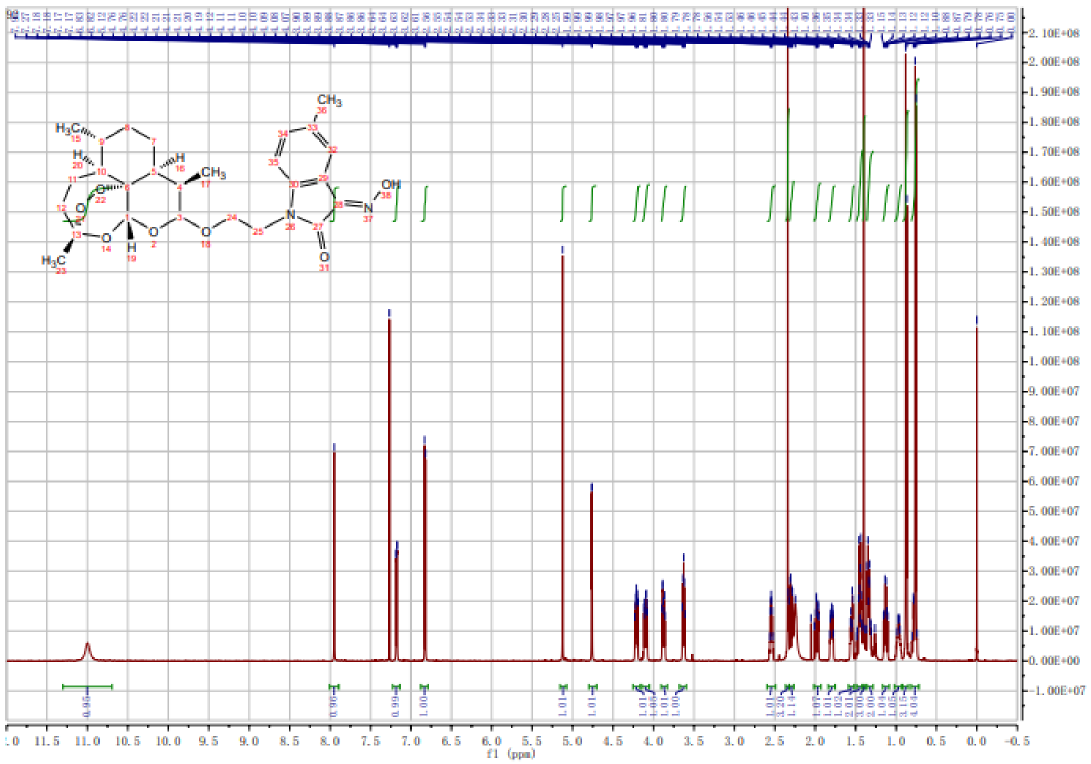

f1 (ppm)

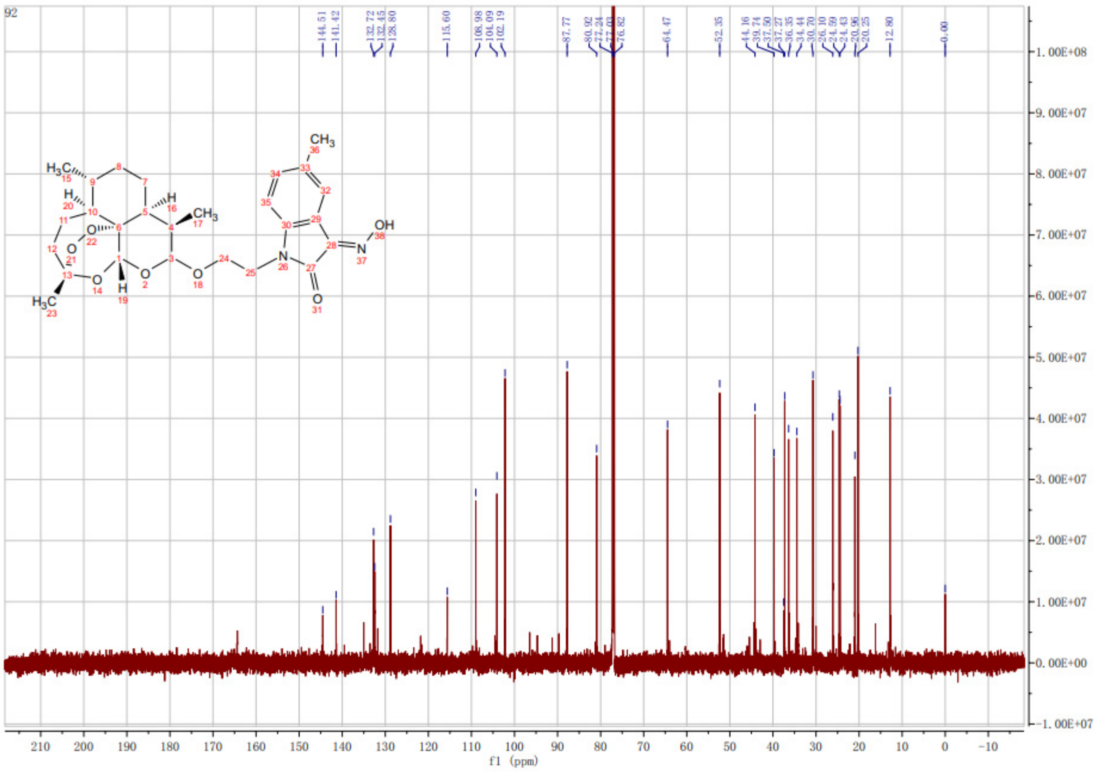

144.51
141.42
132.72
132.45
128.80
115.60
108.98
104.09
102.19
87.77
80.92
77.24
77.03
76.82
64.47
52.35
f1 (ppm)

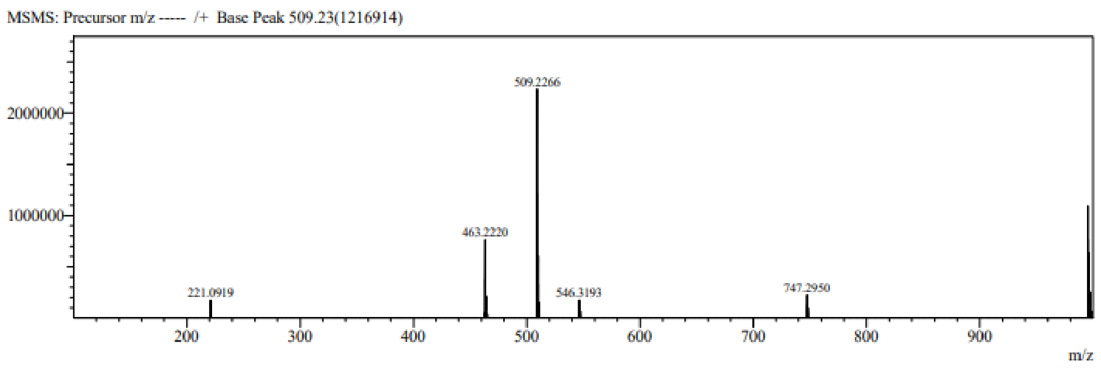

3-(hydroxyimino)-5-methyl-1-(3-(((3R,5aS,6R,8aS,9R,12R,12aR)-3,6,9-trimethyldecahydro-12H-3,12-epoxy[1,2]dioxepino[4,3-i]isochromen-10-yl)oxy)propyl)indolin-2-one (**7b**)

Yellow solid. ^{1}H NMR (600 Hz, DMSO-*d*6) δ 0.84–0.99 (m, 7H), 1.24–1.37 (m, 3H), 1.43–1.59 (m, 5H), 1.64–1.68 (m, 1H), 1.78–1.82 (m, 1H), 1.87–1.98 (m, 2H), 2.01–2.06 (m, 2H), 2.34 (s, 3H, CH3), 2.36–2.44 (m, 1H), 2.64–2.68 (m, 1H), 3.43–3.52 (m, 1H), 3.70–4.14 (m, 3H), 4.81 (d, *J* = 2.0 Hz, 1H), 5.45 (s, 1H), 6.76 (d, J = 4.0 Hz, 1H), 7.16 (d, J = 4.0 Hz, 1H), 7.95 (s, 1H). ^{13}C NMR (150 Hz, DMSO-*d*6) 164.25, 144.55, 141.38, 134.99, 132.77, 132.63, 132.49, 128.96, 128.79, 115.78, 108.84, 108.47, 108.30, 104.32, 104.19, 102.13, 100.30, 91.24, 89.70, 97.97, 81.11, 80.43, 66.29, 65.66, 60.45, 58.65, 52.57, 51.67, 51.44, 45.39, 44.44, 37.48, 37.41, 36.42, 34.66, 30.90, 28.01, 26.16, 24.70, 24.66, 24.57, 20.97, 20.39, 20.29, 13.09, 12.73. HRMS-ESI: m/z Calcd for $C_{27}H_{36}N_2O_7Na$ [M+Na]$^+$: 523.2415; Found: 523.2394.

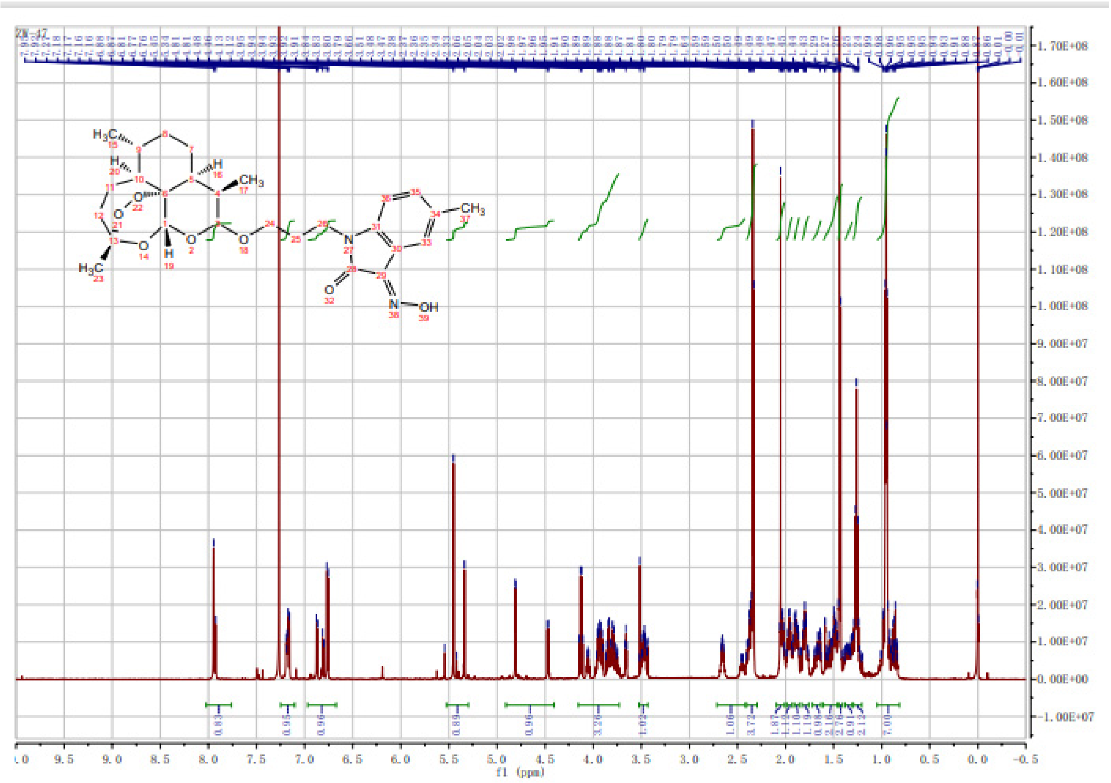

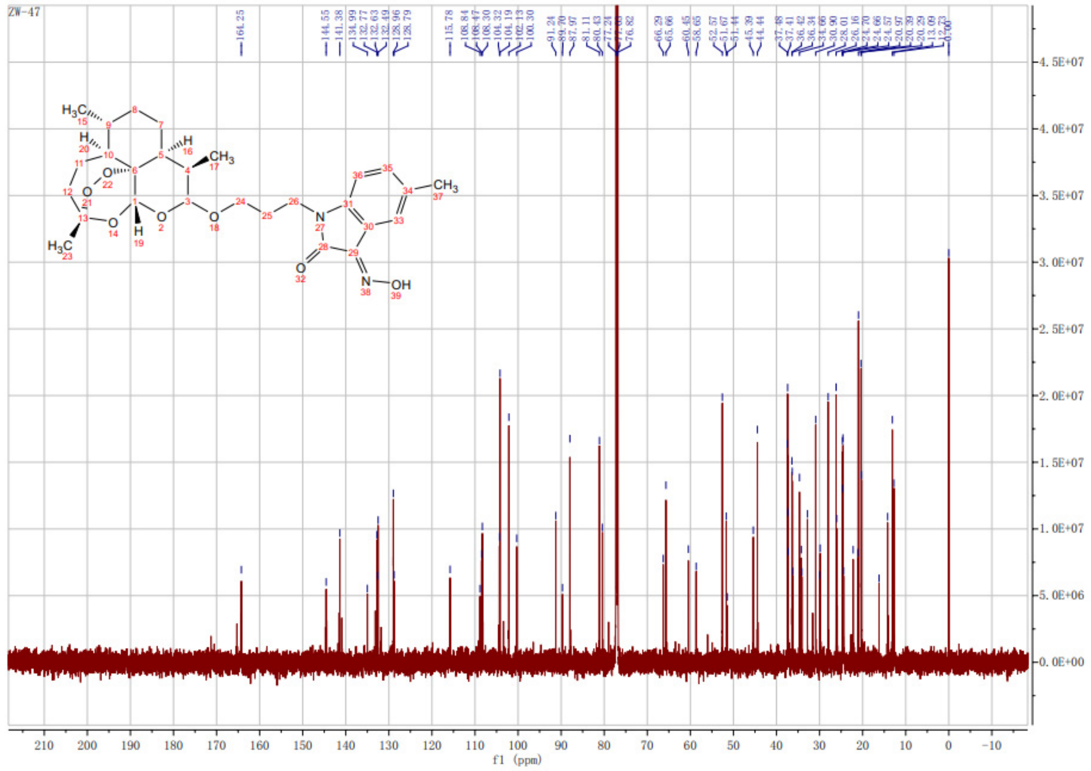

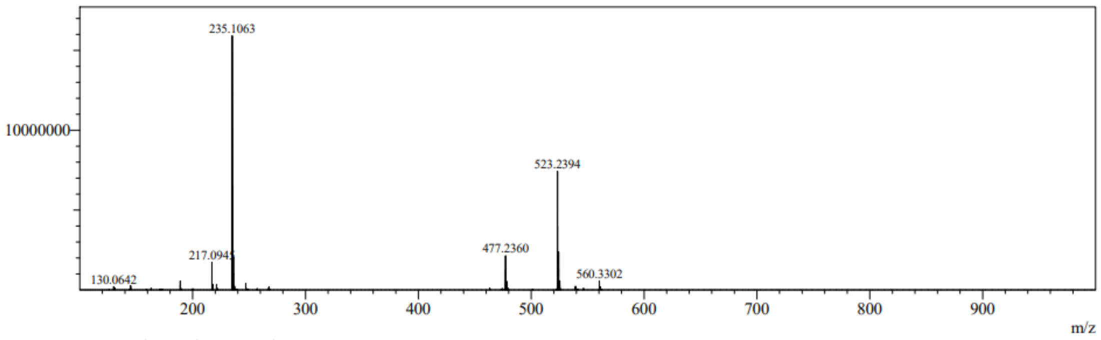

3-(hydroxyimino)-5-methyl-1-(4-(((3R,5aS,6R,8aS,9R,12R,12aR)-3,6,9-trimethyldecahydro-12H-3,12-epoxy[1,2]dioxepino[4,3-i]isochromen-10-yl)oxy)butyl)indolin-2-one (**7c**)

Yellow solid. ^{1}H NMR (600 Hz, $CDCl_3$) 0.84–0.99 (m, 7H), 1.21–1.30 (m, 3H), 1.42–1.53 (m, 5H), 1.58–1.82 (m, 6H), 1.85–1.88 (m, 1H), 2.01–2.05 (m, 1H), 2.31–2.38 (m, 4H), 2.60–2.62 (m, 1H), 3.38–3.41 (m, 1H), 3.73–3.89 (m, 3H), 4.76 (d, *J* = 2.0 Hz, 1H), 5.36 (s, 1H), 6.14 (d, *J* = 4.0 Hz, 1H), 7.18 (d, *J* = 4.0 Hz, 1H), 7.93 (d, *J* = 4.0 Hz, 1H), 10.84 (brs, 1H). ^{13}C NMR (150 Hz, $CDCl_3$) 132.73, 108.39, 104.12, 102.08, 100.05, 91.22, 87.92, 81.11, 67.84, 52.57, 44.42, 39.80, 37.43, 36.43, 34.58, 30.90, 27.20, 26.18, 24.64, 24.50, 24.11, 20.97, 20.36, 20.28, 13.04, 12.64. HRMS-ESI: m/z Calcd for $C_{28}H_{38}N_2O_8Na$ $[M+Na]^+$: 537.2571; Found: 537.2568.

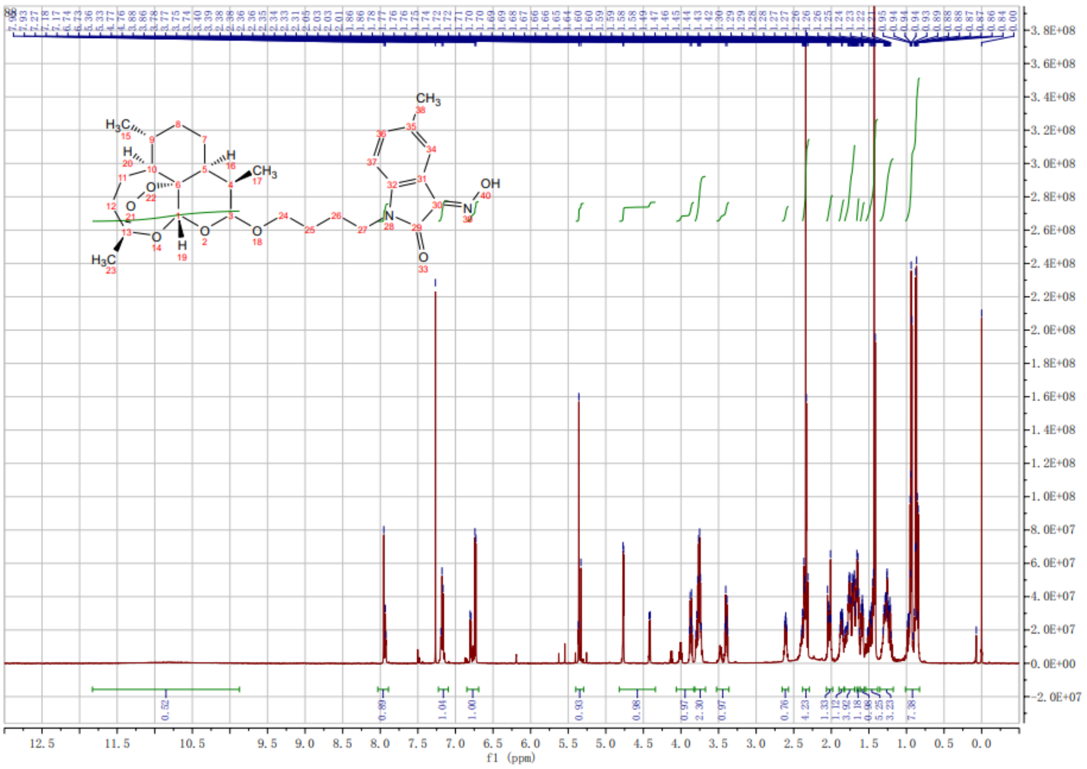
88
f1 (ppm)

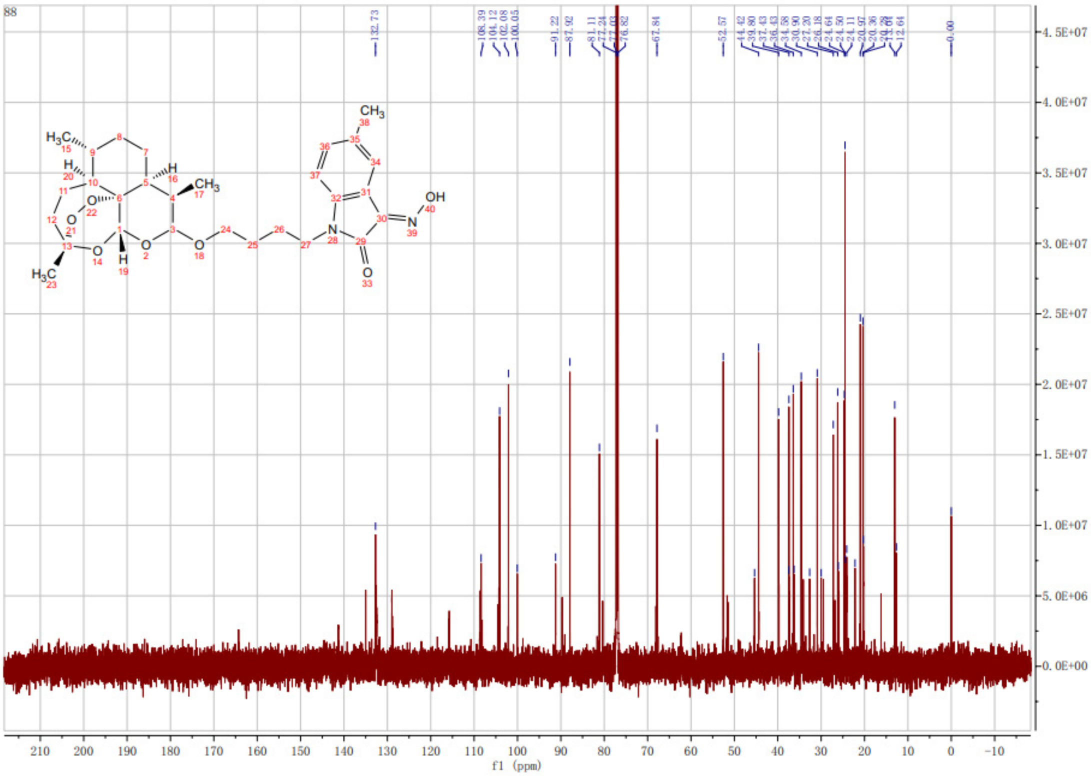
88
f1 (ppm)

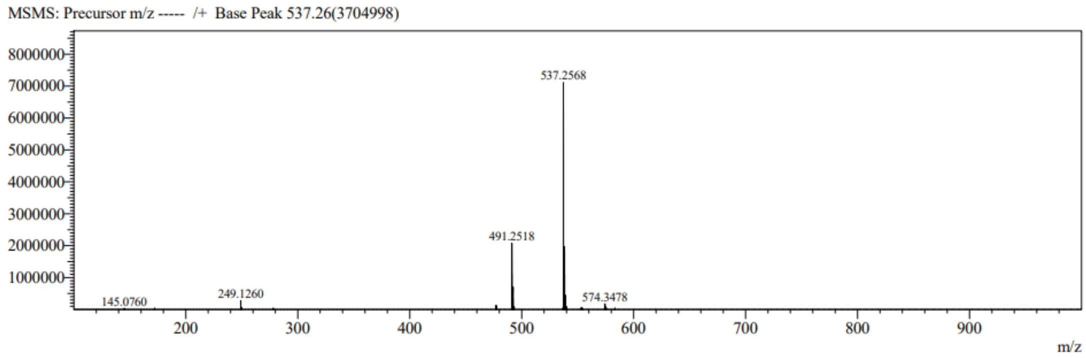

2-(5-methyl-2-oxo-1-(2-(((3R,5aS,6R,8aS,9R,12R,12aR)-3,6,9-trimethyldecahydro-12H-3,12-epoxy[1,2]dioxepino[4,3-i]isochromen-10-yl)oxy)ethyl)indolin-3-ylidene)hydrazine-1-carboxamide (**7d**)

Yellow solid. ^{1}H NMR (600 Hz, $CDCl_3$) 0.75–0.91 (m, 7H), 1.04–1.06 (m, 1H), 1.14–1.18 (m, 1H), 1.36–1.40 (m, 5H), 1.46–1.51 (m, 2H), 1.56–1.59 (m, 1H), 1.81–1.85 (m, 1H), 1.98–2.01 (m, 1H), 2.30–2.35 (m, 4H), 2.55–2.58 (m, 1H), 3.63–3.67 (m, 1H), 3.86–3.90 (m, 1H), 4.00–4.03 (m, 1H), 4.14–4.18 (m, 1H), 4.76 (d, *J* = 2.0 Hz, 1H), 5.19 (s, 1H), 6.86 (d, *J* = 4.0 Hz, 1H), 7.38 (d, *J* = 4.0 Hz, 1H), 7.41 (s, 1H). ^{13}C NMR (150 Hz, $CDCl_3$) 183.47, 158.47, 148.82, 138.52, 133.52, 125.65, 117.58, 110.49, 104.15, 102.42, 87.81, 80.85, 64.61, 52.34, 44.10, 40.04, 37.39, 36.32, 34.40, 30.66, 26.09, 24.62, 24.46, 20.65, 20.25, 12.82.

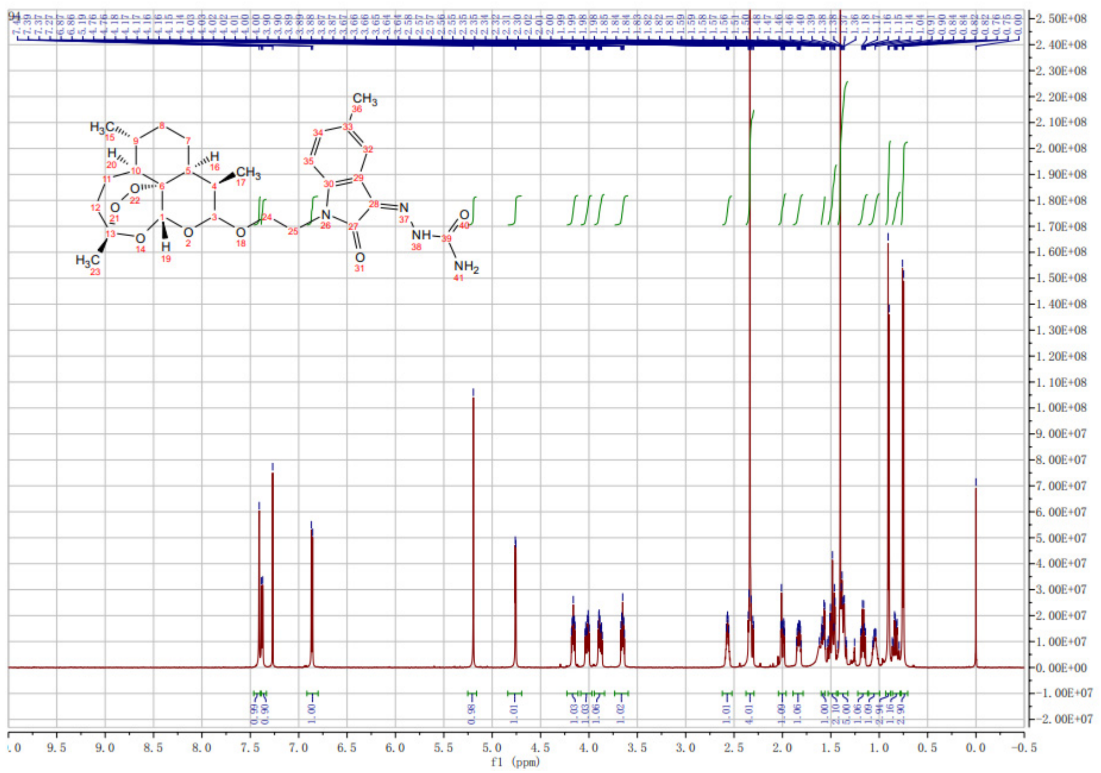

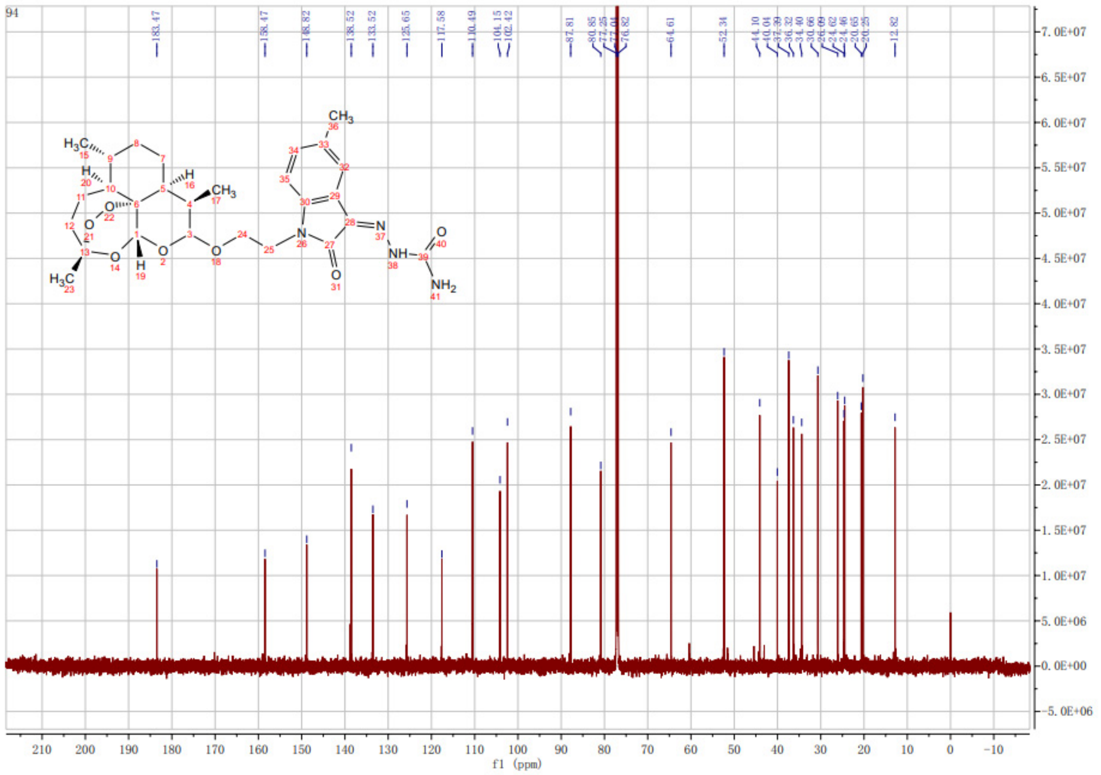

2-(5-methyl-2-oxo-1-(4-(((3R,5aS,6R,8aS,9R,12R,12aR)-3,6,9-trimethyldecahydro-

12H-3,12-epoxy[1,2]dioxepino[4,3-i]isochromen-10-yl)oxy)butyl)indolin-3-ylidene)hydrazine-1-carboxamide (**7e**)

Yellow solid. ^{1}H NMR (600 Hz, CD_3OD) 0.72–0.87 (m, 7H), 1.06–1.14 (m, 2H), 1.22–1.32 (m, 5H), 1.39–1.48 (m, 1H), 1.55–1.77 (m, 7H), 1.90–1.94 (m, 1H), 2.16–2.25 (m, 4H), 2.39–2.42 (m, 1H), 3.36–3.86 (m, 4H), 4.60 (d, *J* = 2.0 Hz, 1H), 5.23 (s, 1H), 6.92 (d, *J* = 4.0 Hz, 1H), 7.30–7.39 (m, 2H). ^{13}C NMR (150 Hz, DMSO-d_6) 187.55, 162.82, 152.73, 142.47, 142.33, 137.44, 128.74, 121.78, 114.21, 107.96, 106.25, 104.00, 95.17, 91.78, 84.71, 84.22, 71.85, 56.47, 48.36, 43.48, 41.17, 39.90, 38.28, 34.92, 28.56, 28.27, 28.24, 28.19, 23.30, 23.14, 15.90, 15.52.

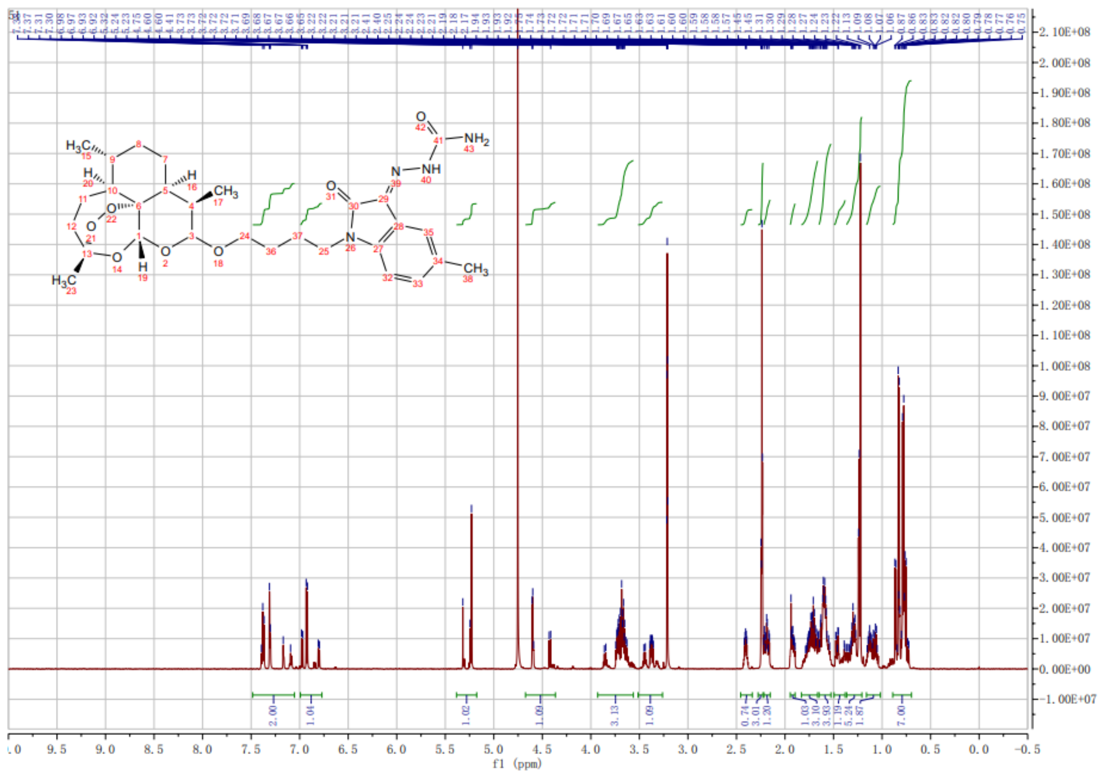

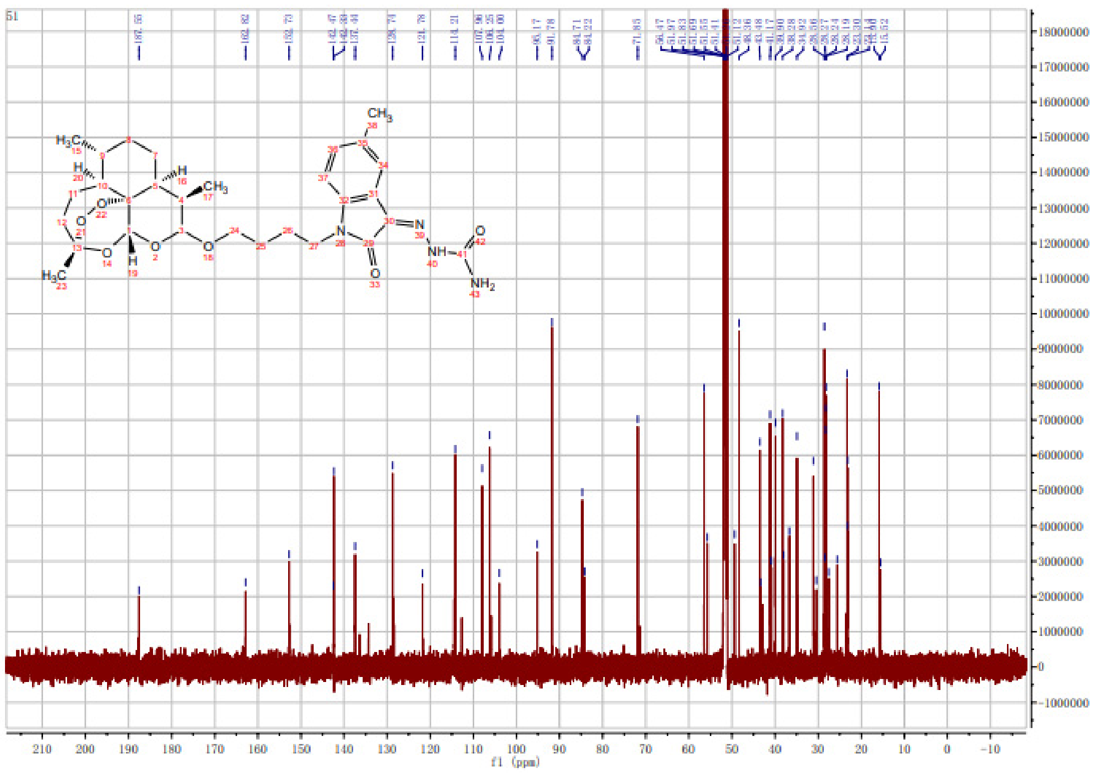

2-(5-methyl-2-oxo-1-(3-(((3R,5aS,6R,8aS,9R,12R,12aR)-3,6,9-trimethyldecahydro-12H-3,12-epoxy[1,2]dioxepino[4,3-i]isochromen-10-yl)oxy)propyl)indolin-3-ylidene)hydrazine-1-carbothioamide (**7f**)

Yellow solid. ^{1}H NMR (600 Hz, CD_3OD) 0.93–1.02 (m, 7H), 1.23–1.34 (m, 2H), 1.40–1.50 (m, 4H), 1.52–1.59 (m, 1H), 1.65–1.73 (m, 2H), 1.77–1.91 (m, 2H), 1.94–2.06 (m, 3H), 2.34–2.39 (m, 4H), 2.64–2.66 (m, 3H), 3.42–3.49 (m, 1H), 3.73–4.05 (m, 3H), 4.80 (d, J = 2.0 Hz, 1H), 5.48 (s, 1H), 6.62 (brs, 1H), 6.80 (d, J = 4.0 Hz, 1H), 7.18 (d, J = 4.0 Hz, 1H), 7.40 (s, 1H), 7.52 (brs, 1H), 12.88 (s, 1H). ^{13}C NMR (150 Hz, DMSO-d_6) 180.01, 161.11, 141.13, 132.91, 132.00, 121.57, 119.44, 109.12, 104.17, 102.17, 87.96, 81.12, 65.34, 52.66, 44.42, 37.35, 37.22, 36.43, 30.90, 27.90, 26.18, 24.69, 24.54, 21.02, 20.47, 13.09, 12.71. HRMS-ESI: m/z Calcd for $C_{28}H_{38}N_4O_6SNa$ [M+Na]$^+$: 581.2404; Found: 581.2377.

HRMS-ESI: m/z Calcd for $C_{28}H_{37}N_4O_6S$ [M-H]$^+$: 577.2439; Found: 577.2428.

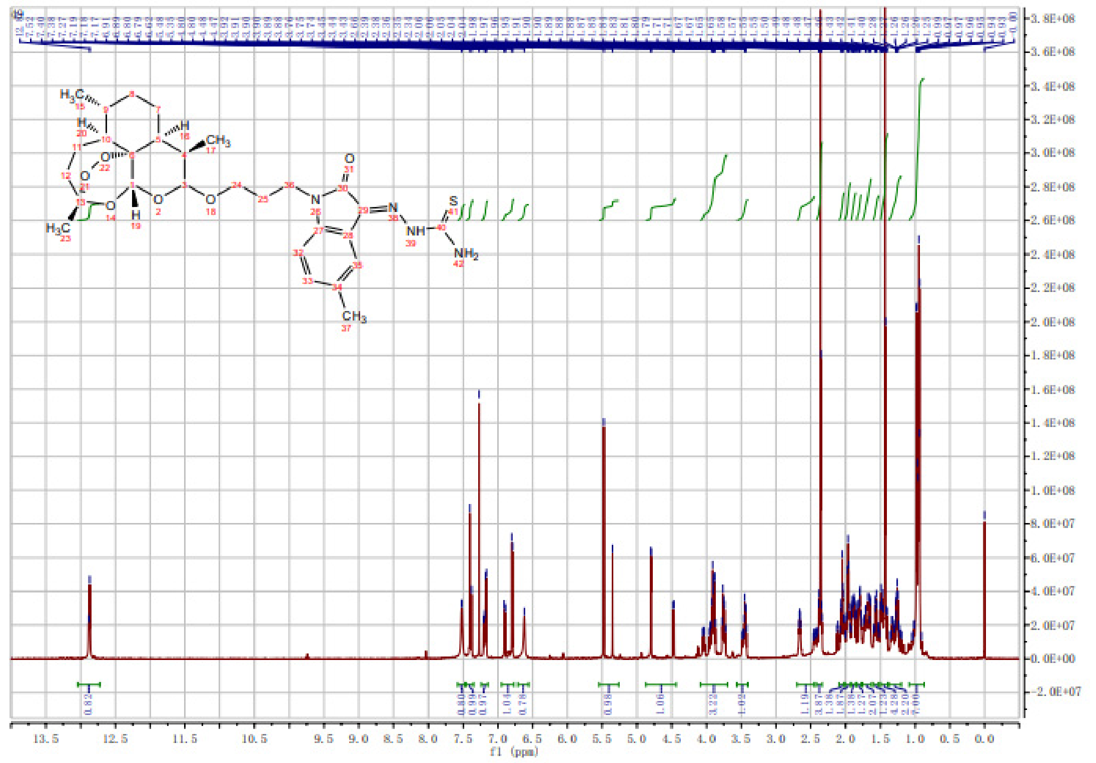

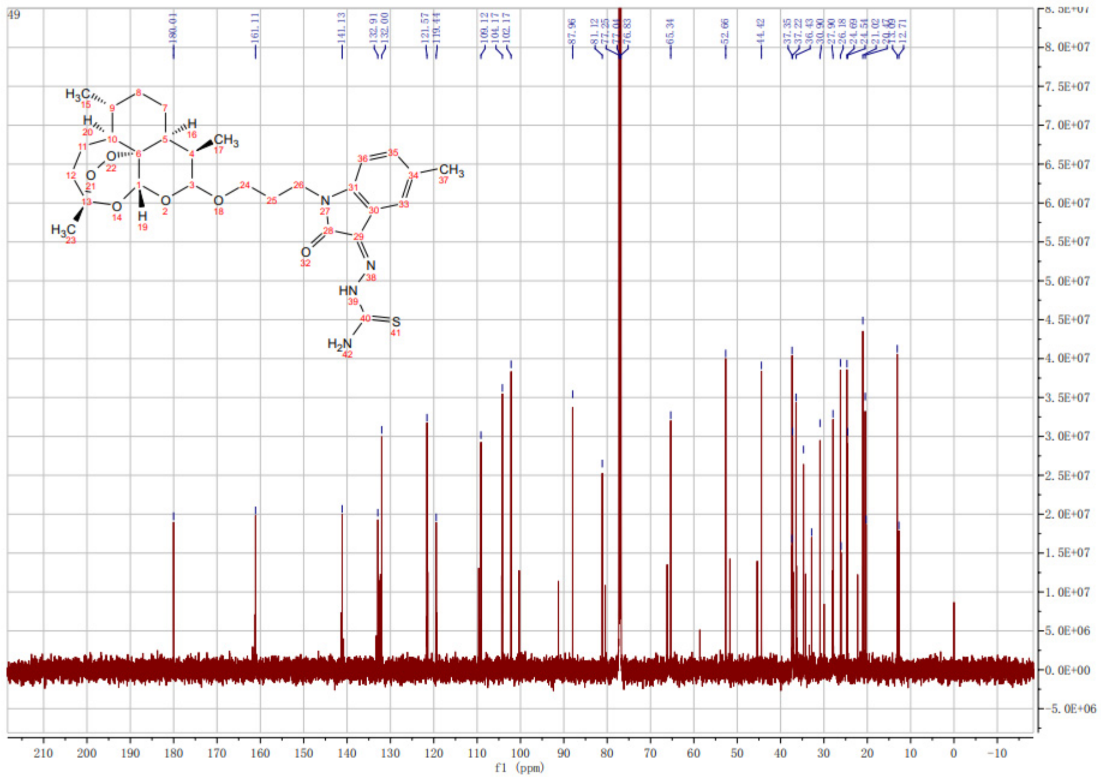

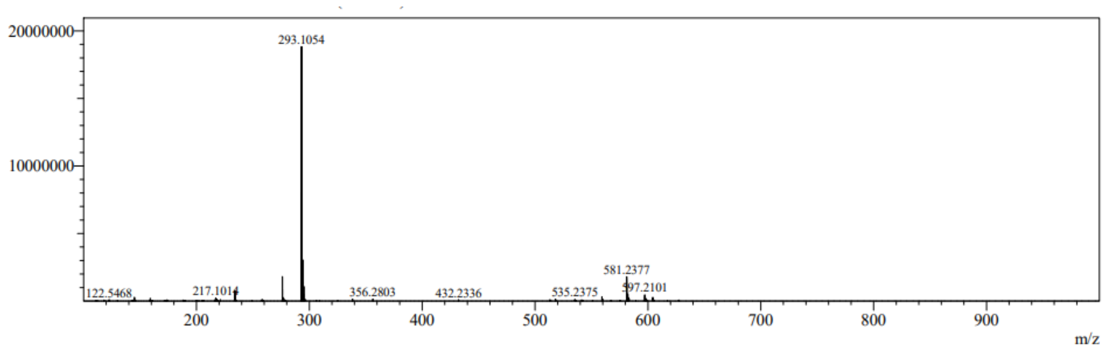

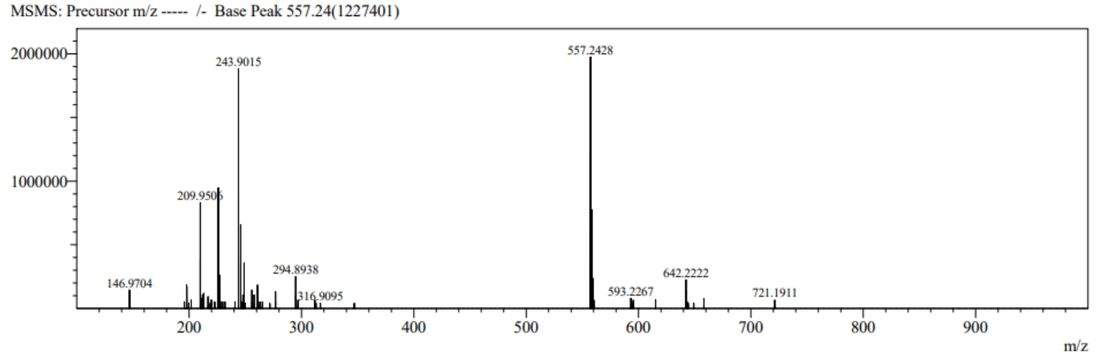

2-(5-methyl-2-oxo-1-(4-(((3R,5aS,6R,8aS,9R,12R,12aR)-3,6,9-trimethyldecahydro-12H-3,12-epoxy[1,2]dioxepino[4,3-i]isochromen-10-yl)oxy)butyl)indolin-3-ylidene)hydrazine-1-carbothioamide (**7g**)

Yellow solid. ^{1}H NMR (600 Hz, DMSO-d_6) 0.76–0.89 (m, 7H), 1.08–1.18 (m, 2H), 1.23–1.34 (m, 5H), 1.40–1.49 (m, 1H), 1.54–1.70 (m, 6H), 1.72–1.77 (m, 1H), 1.95–1.98 (m, 1H), 2.12–2.17 (m, 1H), 2.34–2.36 (m, 1H), 3.36–3.44 (m, 1H), 3.67–3.78 (m, 3H), 4.65 (d, *J* = 2.0 Hz, 1H), 5.21 (s, 1H), 7.06 (d, *J* = d.0 Hz, 1H), 7.22 (d, *J* = 4.0 Hz, 1H), 7.55 (s, 1H), 8.69 (s, 1H), 9.03 (s, 1H), 12.40 (s, 1H). ^{13}C NMR (150 Hz, DMSO-d_6) 183.93, 165.98, 145.97, 137.12, 126.63, 126.41, 126.49, 124.69, 114.93, 108.49, 106.32, 92.15, 85.64, 72.50, 57.27, 49.03, 41.91, 35.73, 30.78, 25.83, 25.44, 17.98. HRMS-ESI: m/z Calcd for $C_{29}H_{40}N_4O_6SNa$ [M+Na]$^{+}$: 595.2561; Found: 595.2525.

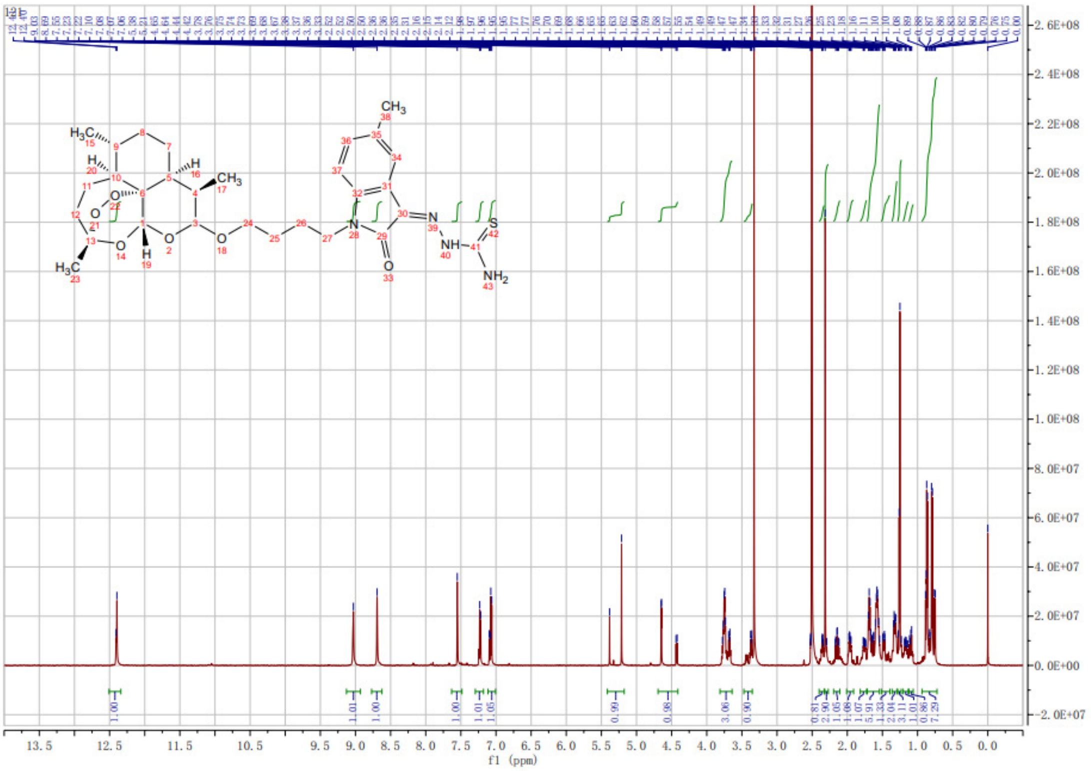
121
f1 (ppm)

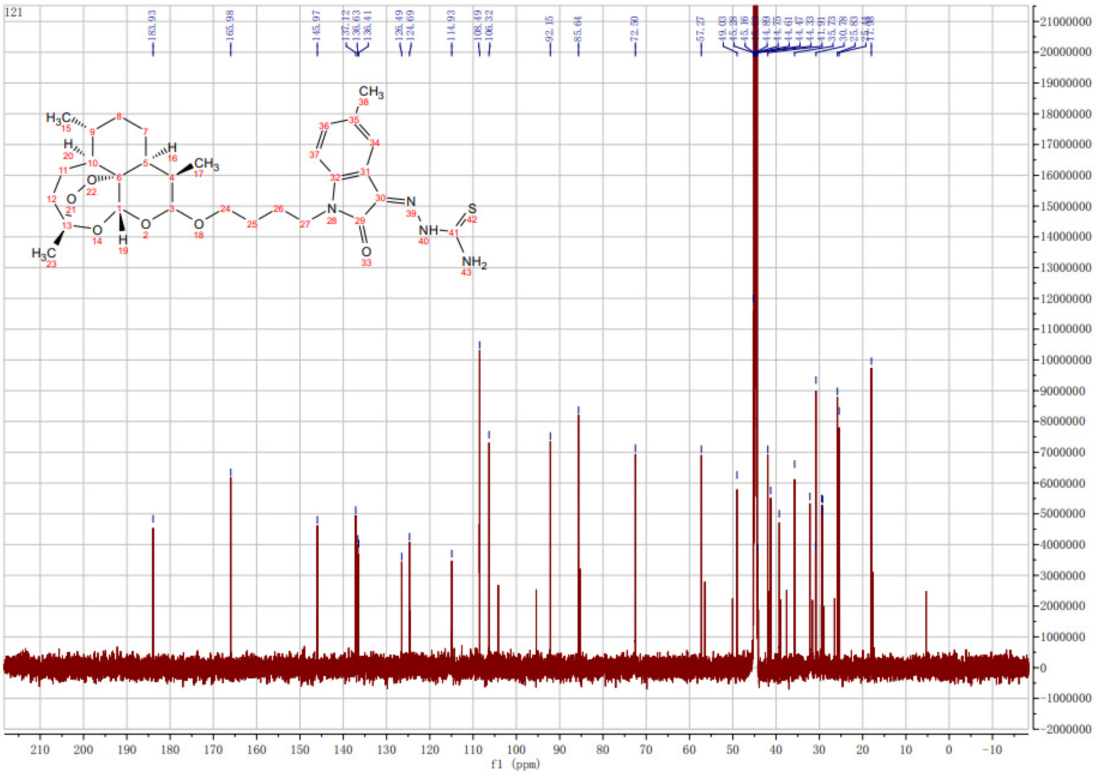
121
183.93
165.98
145.97
126.49
124.69
114.93
108.49
106.32
92.15
85.64
72.50
57.27
f1 (ppm)

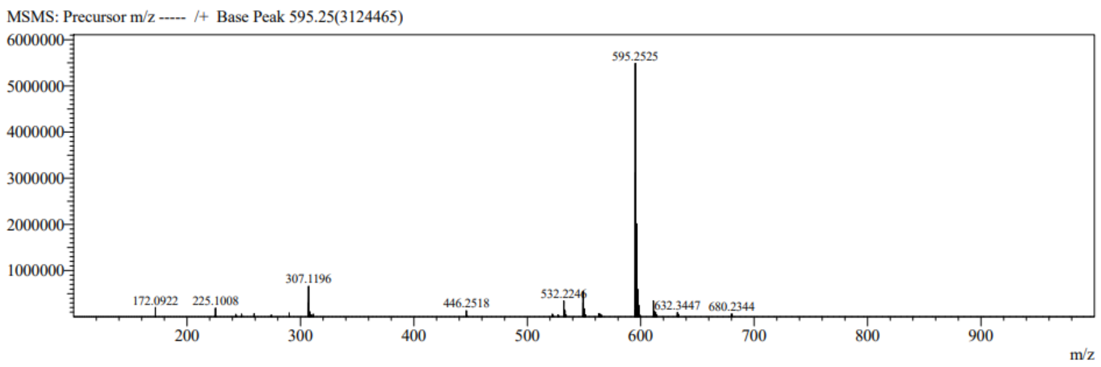

3-(methoxyimino)-1-(4-(((3R,5aS,6R,8aS,9R,12R,12aR)-3,6,9-trimethyldecahydro-12H-3,12-epoxy[1,2]dioxepino[4,3-i]isochromen-10-yl)oxy)butyl)indolin-2-one (**7h**)

Yellow solid. ^{1}H NMR (600 Hz, $CDCl_3$) 0.85–0.96 (m, 7H), 1.20–1.30 (m, 2H), 1.41–1.52 (m, 5H), 1.57–1.78 (m, 7H), 1.84–1.89 (m, 1H), 2.01–2.04 (m, 1H), 2.33–2.38 (m, 1H), 2.59–2.63 (m, 1H), 3.37–3.41 (m, 1H), 3.73–3.82 (m, 2H), 3.85–3.88 (m, 1H), 4.30 (s, 3H), 4.76 (d, *J* = 2.0 Hz, 1H), 5.35 (s, 1H), 6.82 (d, *J* = 8.0 Hz, 1H), 7.06 (t, *J* = 8.0 Hz, 1H), 7.38 (t, *J* = 4.0 Hz, 1H), 7.96 (d, *J* = 4.0 Hz, 1H),. ^{13}C NMR (150 Hz, $CDCl_3$) 163.47, 143.85, 143.61, 132.38, 128.04, 122.82, 115.88, 108.58, 104.08, 102.04, 87.90, 81.10, 67.80, 64.75, 52.57, 44.42, 39.73, 37.43, 36.44, 34.59, 30.89, 27.17, 26.20, 24.65, 24.49, 24.47, 20.35, 13.03. HRMS-ESI: m/z Calcd for $C_{28}H_{38}N_2O_7Na$ $[M+Na]^+$: 537.2555; Found: 537.2571.

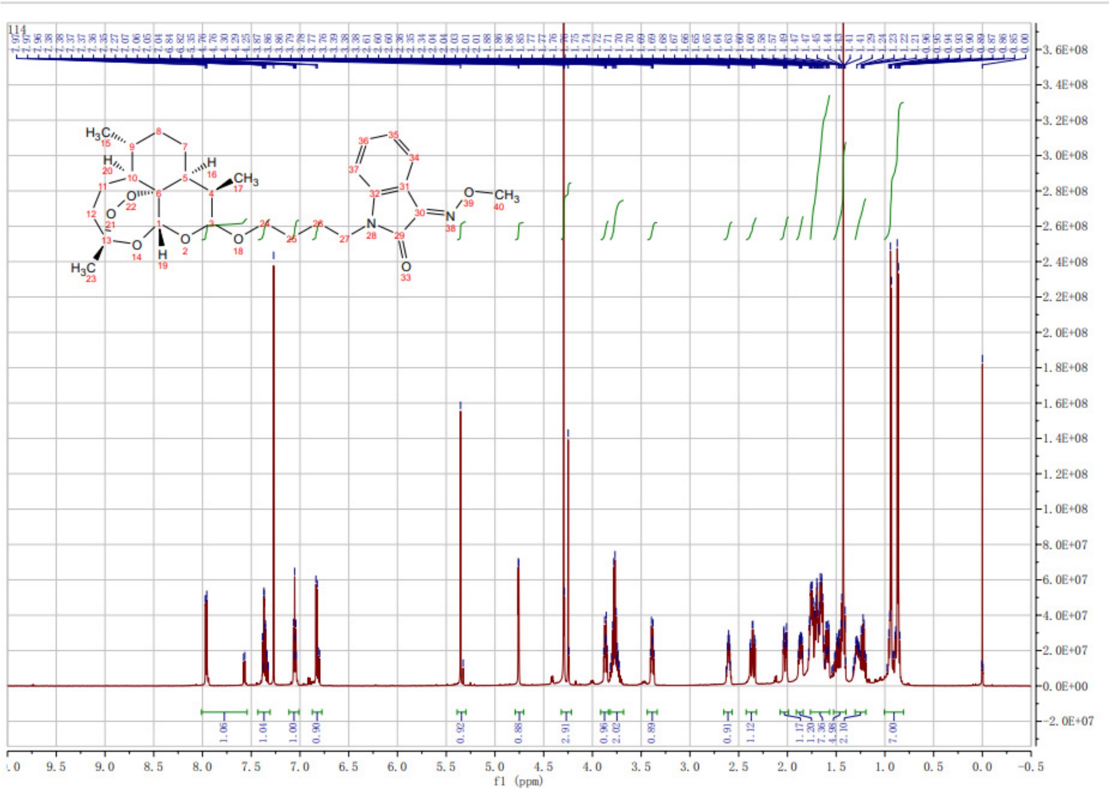

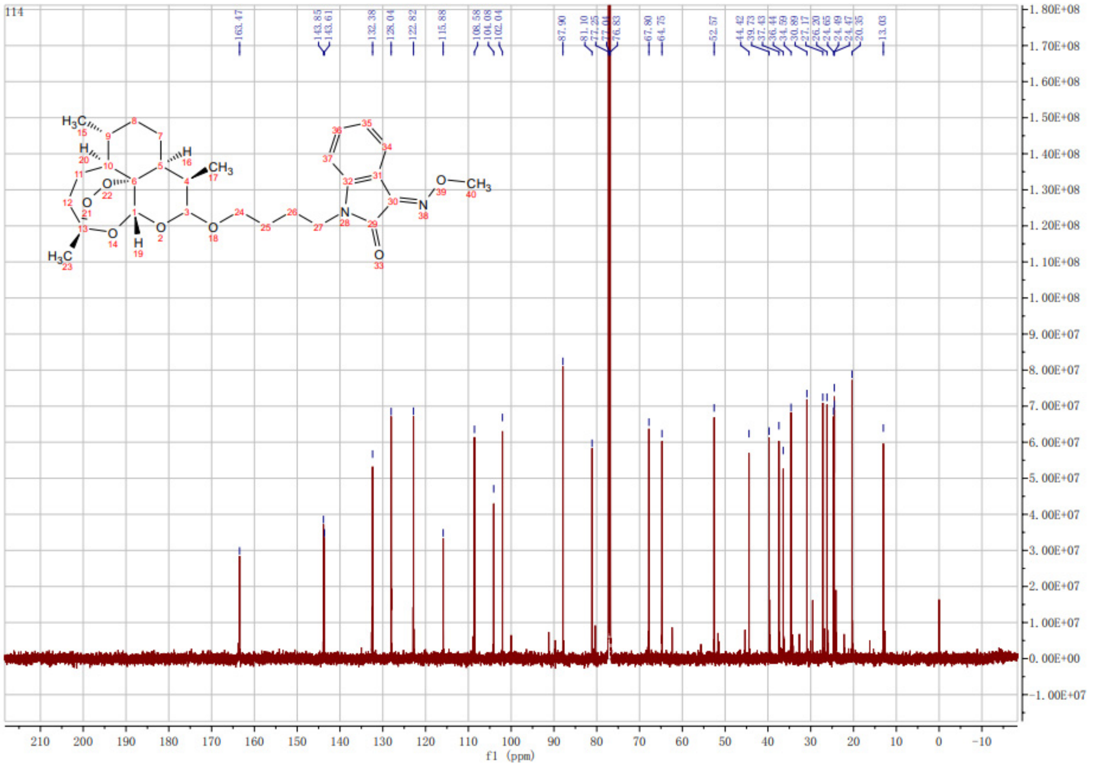

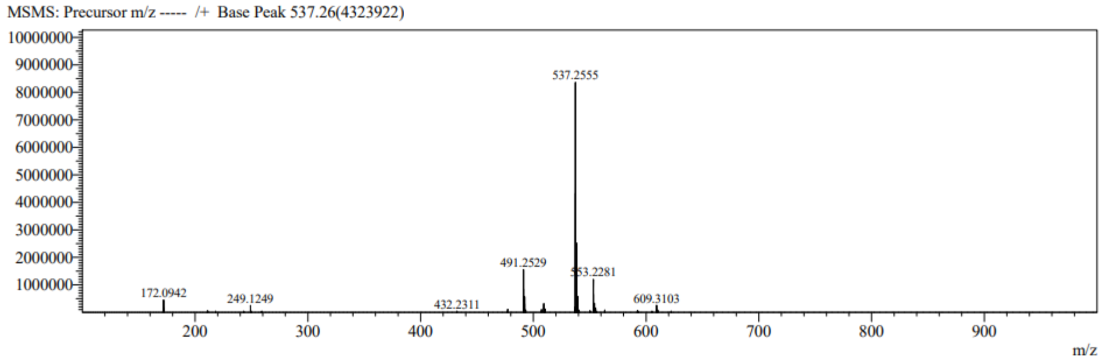

3-(ethoxyimino)-5-methyl-1-(2-(((3R,5aS,6R,8aS,9R,12R,12aR)-3,6,9-trimethyldecahydro-12H-3,12-epoxy[1,2]dioxepino[4,3-i]isochromen-10-yl)oxy)ethyl)indolin-2-one (**7i**)

Yellow solid. ^{1}H NMR (600 Hz, $CDCl_3$) 0.73–0.90 (m, 7H), 0.92–1.00 (m, 1H), 1.33–1.36 (m, 2H), 1.40 (s, 3H), 1.43–1.47 (m, 5H), 1.54–1.57 (m, 1H), 1.79–1.82 (m, 1H), 1.96–2.02 (m, 1H), 2.29–2.32 (m, 1H), 2.34 (s, 3H), 2.52–2.55 (m, 1H), 3.59–3.62 (m, 1H), 3.83–3.87 (m, 1H), 4.07–4.12 (m, 1H), 4.16–4.20 (m, 1H), 4.56 (q, J = 8.0 Hz, 1H), 4.74 (d, J = 4.0 Hz, 1H), 5.12 (s, 1H), 6.80 (d, J = 8.0 Hz, 1H), 7.16 (d, J = 8.0 Hz, 1H), 7.78 (s, 1H). ^{13}C NMR (150 Hz, $CDCl_3$) 163.79, 143.53, 141.54, 132.37, 132.30, 128.38, 115.85, 108.88, 104.03, 102.18, 87.73, 80.90, 72.94, 64.52, 52.36, 44.18, 39.59, 37.26, 36.36, 34.47, 30.71, 26.11, 24.60, 24.42, 21.00, 20.25, 14.80, 12.80. HRMS-ESI: m/z Calcd for $C_{27}H_{39}N_2O_7$ $[M+H_3O]^+$: 519.2701; Found: 519.2194.

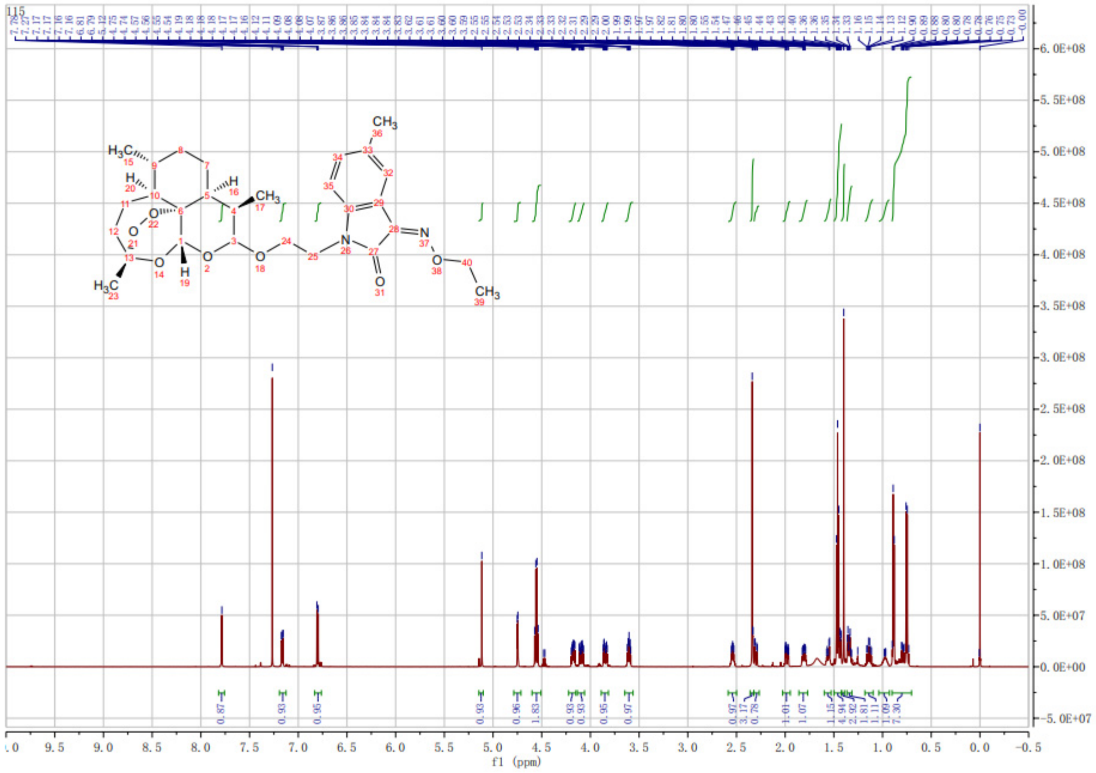

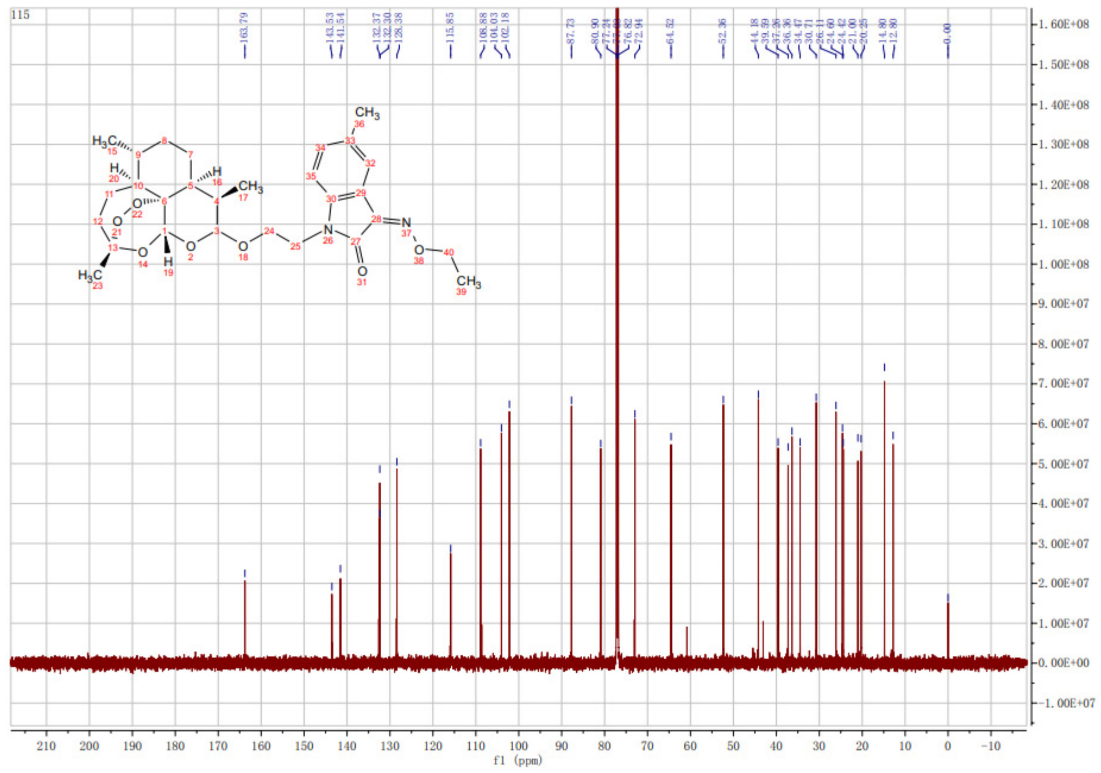

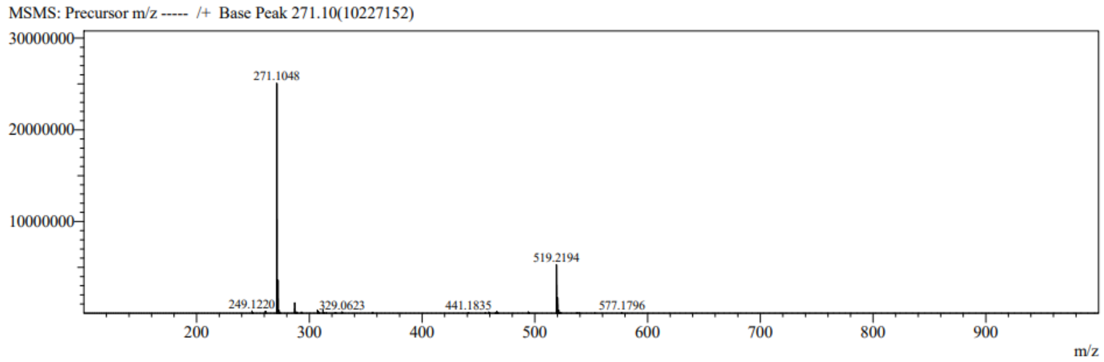

3-(ethoxyimino)-5-methoxy-1-(4-(((3R,5aS,6R,8aS,9R,12R,12aR)-3,6,9-trimethyldecahydro-12H-3,12-epoxy[1,2]dioxepino[4,3-i]isochromen-10-yl)oxy)butyl)indolin-2-one (**7j**)

Yellow solid. ^{1}H NMR (600 Hz, $CDCl_3$) 0.84–0.96 (m, 7H), 1.20–1.30 (m, 2H), 1.41–1.51 (m, 7H), 1.58–1.66 (m, 4H), 1.68–1.77 (m, 4H), 1.85–1.88 (m, 1H), 2.01–2.04 (m, 1H), 2.33–2.38 (m, 4H), 2.59–2.62 (m, 1H), 3.36–3.40 (m, 1H), 3.71–3.78 (m, 2H), 3.84–3.88 (m, 1H), 4.56 (q, J = 4.0 Hz, 1H), 4.76 (d, J = 4.0 Hz, 1H), 5.35 (s, 1H), 6.72 (d, J = 4.0 Hz, 1H), 7.17 (d, J = 4.0 Hz, 1H), 7.80 (s, 1H). ^{13}C NMR (150 Hz, $CDCl_3$) 163.63, 143.64, 141.52, 132.48, 132.30, 128.56, 108.28, 104.04, 102.04, 87.90, 81.11, 72.94, 67.85, 52.58, 44.43, 39.70, 37.43, 36.45, 34.59, 30.91, 27.17, 26.21, 24.65, 24.50, 21.01, 20.36, 14.80, 13.04. HRMS-ESI: m/z Calcd for $C_{30}H_{42}N_2O_7Na$ $[M+Na]^+$: 565.2884; Found: 565.2850.

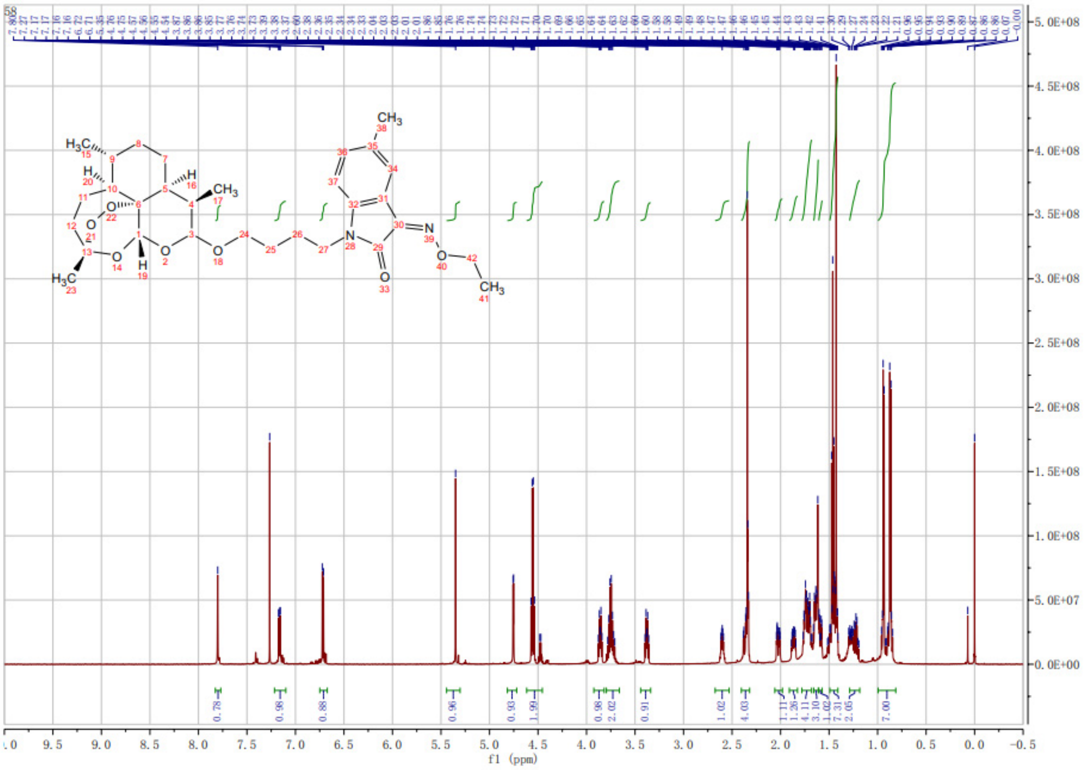
f1 (ppm)

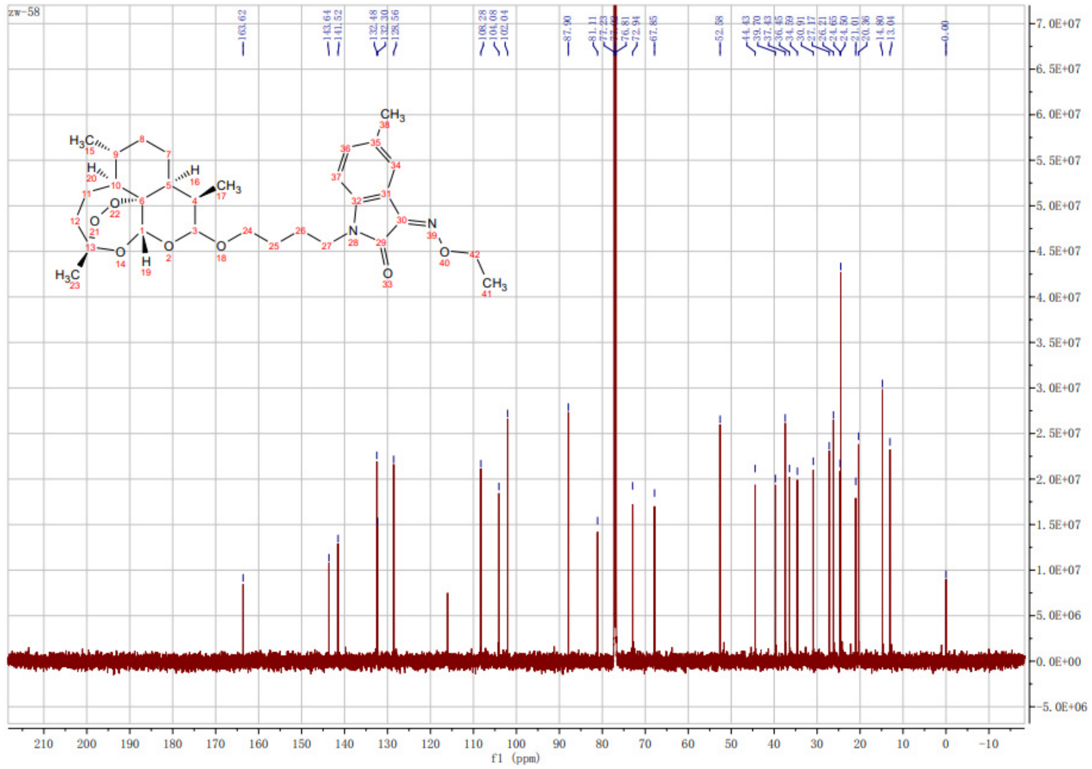
zw-58
f1 (ppm)

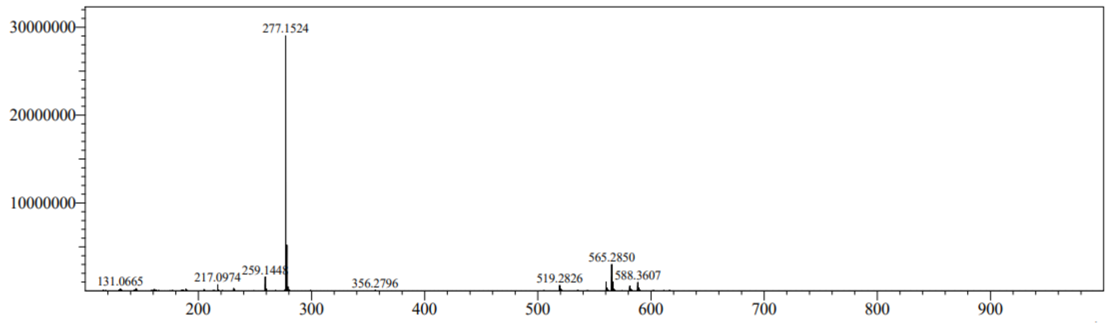

3-((benzyloxy)imino)-5-methyl-1-(2-(((3R,5aS,6R,8aS,9R,12R,12aR)-3,6,9-trimethyldecahydro-12H-3,12-epoxy[1,2]dioxepino[4,3-i]isochromen-10-yl)oxy)ethyl)indolin-2-one (**7k**)

Yellow solid. ^{1}H NMR (600 Hz, $CDCl_3$) 0.74–0.88 (m, 7H), 0.95–0.99 (m, 1H), 1.11–1.16 (m, 1H), 1.31–1.36 (m, 2H), 1.40–1.50 (m, 5H), 1.52–1.57 (m, 1H), 1.78–1.82 (m, 1H), 1.97–2.00 (m, 1H), 2.29 (s, 3H), 2.31–2.34 (m, 1H), 2.52–2.55 (m, 1H), 3.59–3.62 (m, 1H), 3.83–3.87 (m, 1H), 4.06–4.09 (m, 1H), 4.14–4.18 (m, 1H), 4.74 (d, J = 2.0 Hz, 1H), 5.13 (s, 1H), 5.25 (s, 2H), 6.80 (d, J = 4.0 Hz, 1H), 7.16 (d, J = 4.0 Hz, 1H), 7.34–7.47 (m, 5H), 7.75 (s, 1H). ^{13}C NMR (150 Hz, $CDCl_3$) 163.67, 144.04, 141.67, 136.43, 132.60, 132.40, 128.61, 128.59, 128.42, 128.37, 128.32, 115.79, 108.93, 104.04, 102.22, 87.74, 80.91, 79.30, 64.55, 52.35, 44.18, 39.64, 37.27, 36.36, 34.45, 30.71, 26.12, 24.61, 24.42, 20.99, 20.24, 12.81. HRMS-ESI: m/z Calcd for $C_{33}H_{40}N_2O_7Na$ $[M+Na]^+$: 599.2728; Found: 599.2715.

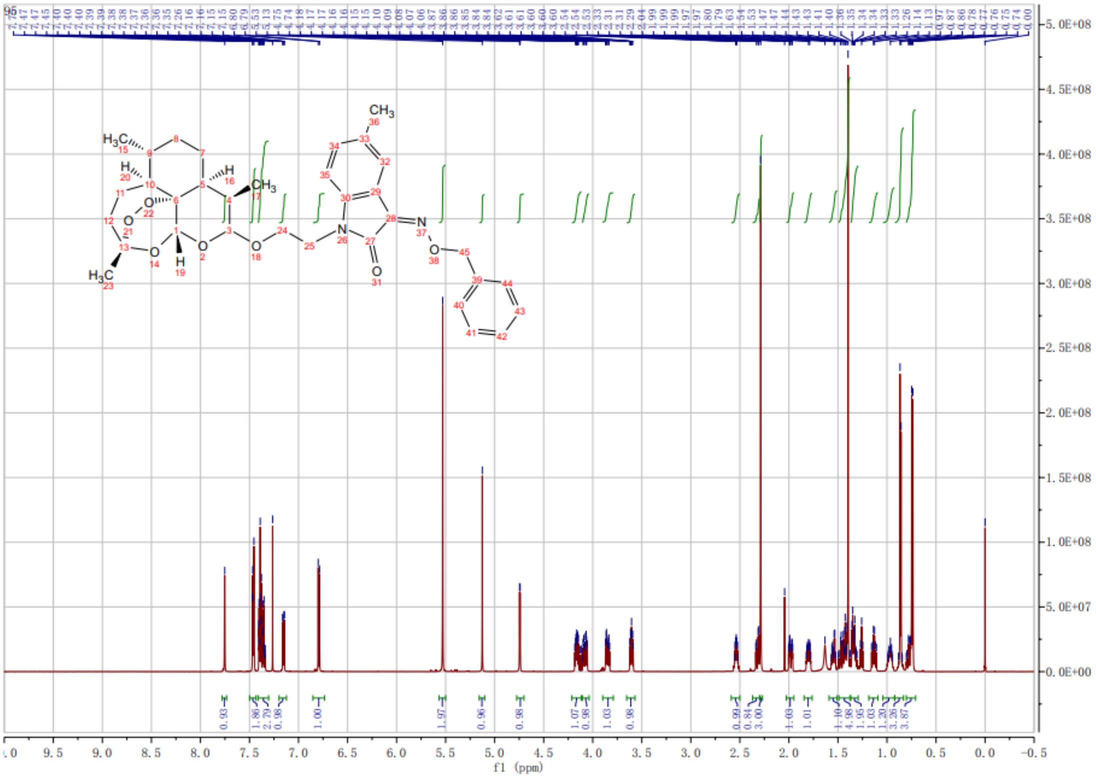

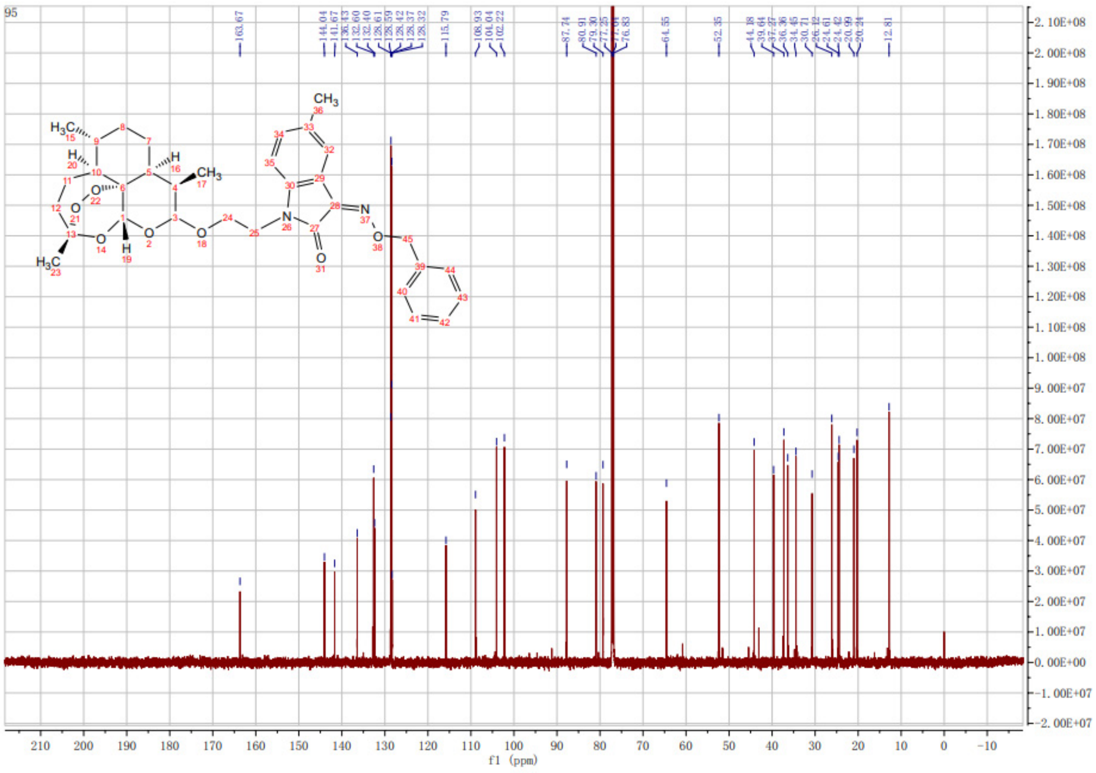

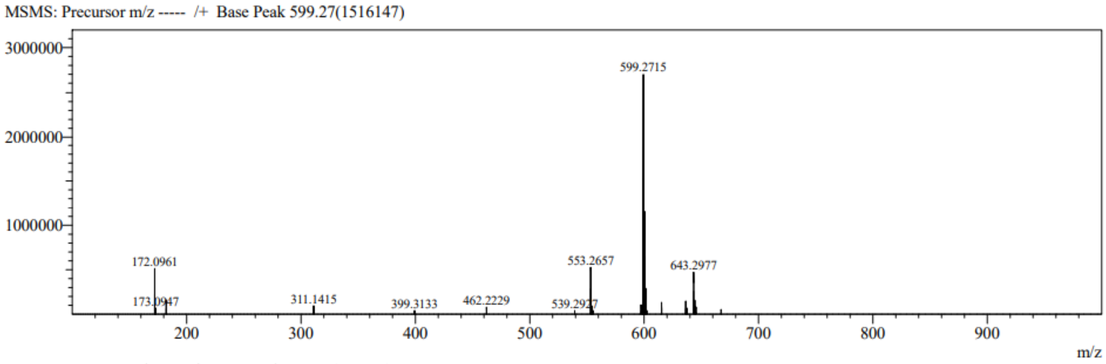

3-((benzyloxy)imino)-6-methyl-1-(3-(((3R,5aS,6R,8aS,9R,12R,12aR)-3,6,9-trimethyldecahydro-12H-3,12-epoxy[1,2]dioxepino[4,3-i]isochromen-10-yl)oxy)propyl)indolin-2-one (**7l**)

This compound was obtained directly from the collaborators and the detailed synthesis method, together with the characterization data have been reported in Literature [21].

3-((benzyloxy)imino)-5-methyl-1-(4-(((3R,5aS,6R,8aS,9R,12R,12aR)-3,6,9-

trimethyldecahydro-12H-3,12-epoxy[1,2]dioxepino[4,3-i]isochromen-10-yl)oxy)butyl)indolin-2-one (**7m**)

Yellow solid. ^{1}H NMR (600 Hz, $CDCl_3$) 0.84–0.98 (m, 7H), 1.22–1.30 (m, 3H), 1.41–1.52 (m, 5H), 1.57–1.77 (m, 6H), 1.85–1.88 (m, 1H), 2.00–2.04 (m, 1H), 2.29 (s, 3H), 2.33–2.38 (m, 1H), 2.59–2.61 (m, 1H), 3.36–3.40 (m, 1H), 3.69–3.88 (m, 3H), 4.76 (d, J = 2.0 Hz, 1H), 5.35 (s, 1H), 5.53 (s, 2H), 6.72 (d, J = 8.0 Hz, 1H), 7.16 (d, J = 8.0 Hz, 1H), 7.35–7.47 (m, 2H), 7.77 (s, 1H). ^{13}C NMR (150 Hz, $CDCl_3$) 163.50, 144.17, 141.65, 136.48, 132.73, 132.40, 128.80, 128.60, 128.40, 128.34, 128.30, 115.95, 108.33, 104.08, 102.05, 87.91, 81.11, 79.26, 67.84, 52.58, 51.69, 44.43, 39.75, 37.42, 36.45, 34.60, 30.91, 27.18, 26.21, 24.66, 24.49, 21.00, 20.36, 13.05. HRMS-ESI: m/z Calcd for $C_{35}H_{44}N_2O_8Na$ $[M+Na]^+$: 627.3041; Found: 627.3032.

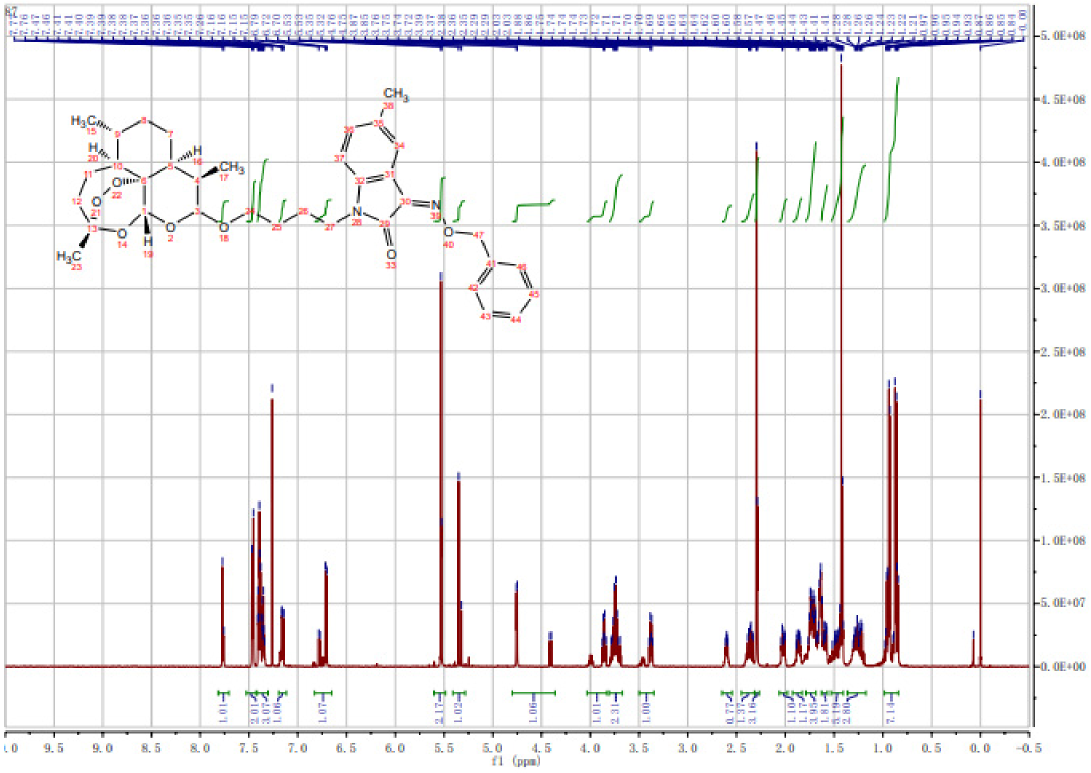

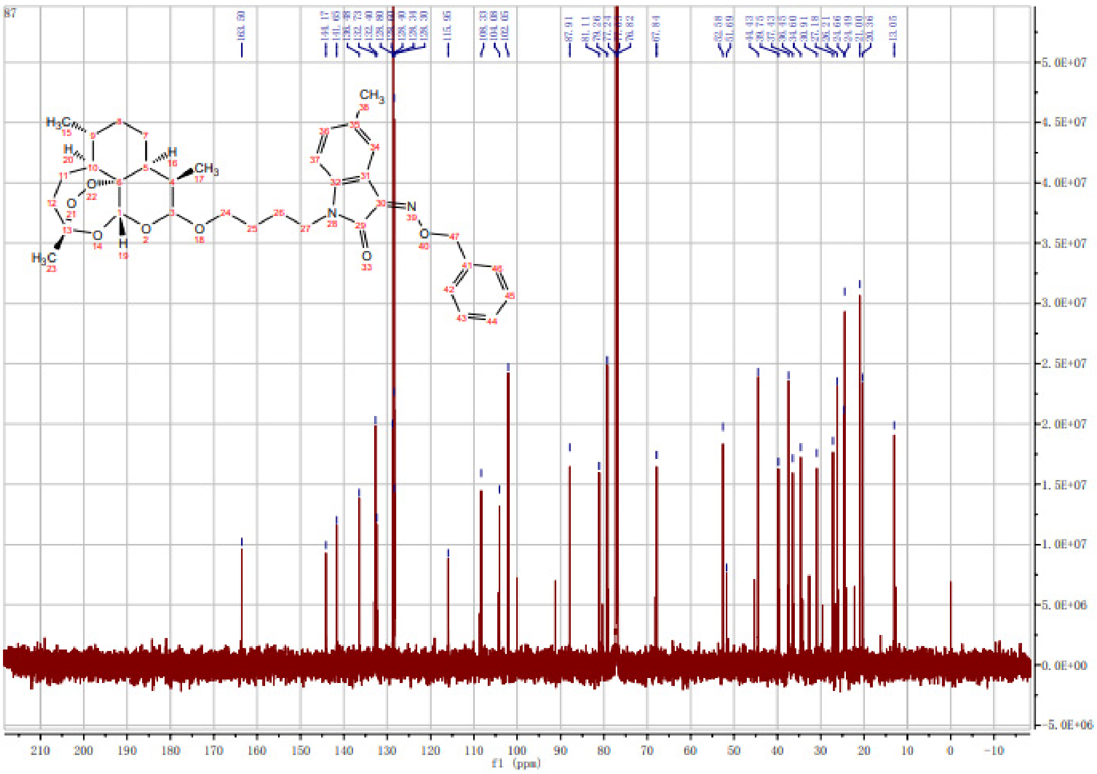

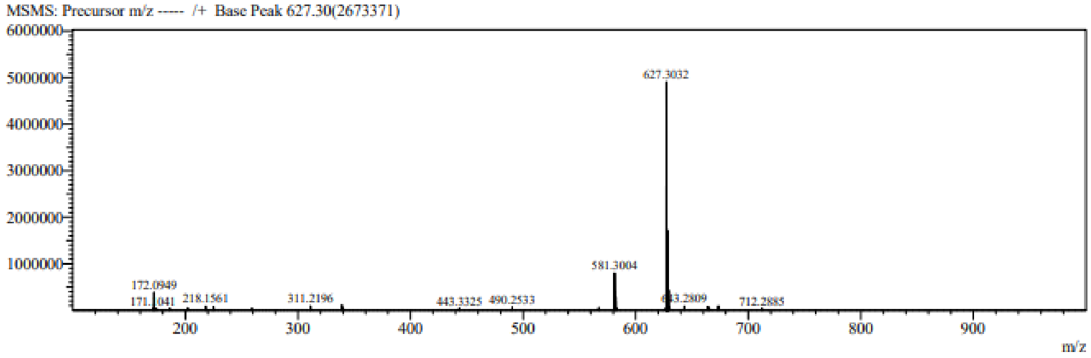

Supplement: Supplementary file 1 [file molecules-27-07994-s001.zip › molecules-2022063-supplementary.pdf]
